# Gut and Gill-Associated Microbiota of the Flatfish European Plaice (*Pleuronectes platessa*): Diversity, Metabolome and Bioactivity against Human and Aquaculture Pathogens

**DOI:** 10.3390/md20090573

**Published:** 2022-09-09

**Authors:** Marjan Ghotbi, Ole Kelting, Martina Blümel, Deniz Tasdemir

**Affiliations:** 1 GEOMAR Centre for Marine Biotechnology (GEOMAR-Biotech), Research Unit Marine Natural Product Chemistry, GEOMAR Helmholtz Centre for Ocean Research Kiel, Am Kiel-Kanal 44, 24106 Kiel, Germany; 2 Faculty of Mathematics and Natural Sciences, Kiel University, Christian-Albrechts-Platz 4, 24118 Kiel, Germany

**Keywords:** European plaice, *Pleuronectes platessa*, microbiota, gut, gill, feature-based molecular networking, fish pathogen, ESKAPE, bioactivity, untargeted metabolomics

## Abstract

Similar to other marine holobionts, fish are colonized by complex microbial communities that promote their health and growth. Fish-associated microbiota is emerging as a promising source of bioactive metabolites. *Pleuronectes platessa* (European plaice, plaice), a flatfish with commercial importance, is common in the Baltic Sea. Here we used a culture-dependent survey followed by molecular identification to identify microbiota associated with the gills and the gastrointestinal tract (GIT) of *P. platessa*, then profiled their antimicrobial activity and metabolome. Altogether, 66 strains (59 bacteria and 7 fungi) were isolated, with Proteobacteria being the most abundant phylum. Gill-associated microbiota accounted for higher number of isolates and was dominated by the Proteobacteria (family Moraxellaceae) and Actinobacteria (family Nocardiaceae), whereas Gram-negative bacterial families Vibrionaceae and Shewanellaceae represented the largest group associated with the GIT. The EtOAc extracts of the solid and liquid media cultures of 21 bacteria and 2 fungi representing the diversity of cultivable plaice-associated microbiota was profiled for their antimicrobial activity against three fish pathogens, human bacterial pathogen panel (ESKAPE) and two human fungal pathogens. More than half of all tested microorganisms, particularly those originating from the GIT epithelium, exhibited antagonistic effect against fish pathogens (*Lactococcus garvieae, Vibrio ichthyoenteri*) and/or human pathogens (*Enterococcus faecium*, methicillin-resistant *Staphylococcus aureus*). Proteobacteria represented the most active isolates. Notably, the solid media extracts displayed higher activity against fish pathogens, while liquid culture extracts were more active against human pathogens. Untargeted metabolomics approach using feature-based molecular networking showed the high chemical diversity of the liquid extracts that contained undescribed clusters. This study highlights plaice-associated microbiota as a potential source of antimicrobials for the control of human and the aquaculture-associated infections. This is the first study reporting diversity, bioactivity and chemical profile of culture-dependent microbiota of plaice.

## 1. Introduction

The marine realm harbors a plethora of microorganisms showing various ecological adaptations and intimate interactions with multicellular organisms [1]. These symbiotic interactions underpin functioning of ecosystems, affect microbial dispersal and maintain microbial diversity [2,3]. This relationship also has tremendous impact on development, homeostasis and protection of the host [4,5,6]. Fish are a group of cold-blooded aquatic vertebrates (phylum Chordata) that breathe with gills [7]. They represent a unique holobiont since their mucosal surfaces, namely skin, gills and gut are in constant and dynamic interactions with the surrounding sea water and the microorganisms therein. Thus, over millenia they have adopted strategies and molecular machinery that enable them to eliminate pathogens while allowing settlement of commensals for their benefit [8]. Commensal microbes are capable of creating “colonization resistance” by outcompeting pathogens for space and nutrients, thereby mitigating infections [9]. Overall, the coevolution of microbial symbionts with their multicellular fish host led them to acquire unique biochemical traits that make them promising sources for novel bioactive molecules [10]. With 33,000 known species [11], fish account for 50% of all vertebrate species [12]. However, fish-associated microbiota has so far received limited attention for their community composition, chemical constituents or their application potentials in different sectors, such as medicine and aquaculture.

European plaice (*Pleuronectes platessa* L., family Pleuronectidae) is a species of marine flatfish commonly found in the North-East Atlantic Ocean and its adjacent seas, namely the North and Baltic Seas [13]. It has an oval shape body characterized by orange spots on its dark-colored dorsal skin and having both eyes on the dorsal side. They are mostly nocturnal and feed on bottom-living animals, such as polychaetes, crustaceans and bivalve mollusks. Population-genetic studies of Northern European plaice based on microsatellite analysis showed spatial as well as temporal homogeneity across large scales [14]. Being a commercially important and popular flatfish, plaice and its application potential beyond food have been investigated. To this end, plaice protein hydrolysates have been shown to inhibit proliferation of breast cancer cell lines [15]. Although its microbiota has been largely unstudied, some reports on plaice-associated microbiota exist: an early culture-based study reported the genera *Pseudomonas*, *Achromobacter*, *Alcaligenes*, *Agrobacterium*, *Vibrio*, *Aeromonas*, *Enterobacteriaceae* and *Moraxella* from plaice skin and additionally *Acinetobacter*, *Staphylococcus*, *Clostridium*, *Flavobacterium*, *Corynebacterium*, *Bacillus*, *Micrococcus*, members of the family Bacteroidaceae, and yeasts from the intestines of farmed plaice [16]. A more recent cultivation-independent report retrieved 304 bacterial genera from plaice gills with sequence reads dominated by the alphaproteobacterial genus *Sphingomonas* and prevalence of several potential pathogenic genera, such as *Shewanella, Pseudoalteromonas*, *Hafnia, Halomonas, Pasteurella, Pseudomonas, Acinetobacter, Francisella, Vibrio, Listonella, Photobacterium, Mycoplasma, Corynebacterium, Micrococcus, Chryseobacterium, Flavobacterium, Tenacibaculum, Staphylococcus, Bacillus, Lactococcus, Streptococcus* and *Clostridium* [17]. The gut of young wild plaice sampled in Scotland was reportedly dominated by the phyla Proteobacteria, Spirochaetes and Tenericutes with a surprising dominance of Alphaproteobacteria and gut microbial community composition varied depending on the plaice body size, nutritional condition and diet [18]. However, to the best of our knowledge, nothing is known on the potential of plaice-associated microorganisms for production of bioactive secondary metabolites. In continuation of our research into culture-dependent microbiota of marine organisms, we investigated, comparatively, the microbial communities associated with the gastrointestinal tract (GIT) and the gills of the Baltic Sea plaice by a culture-dependent approach, which was followed by the antimicrobial activity assessments and Liquid Chromatography–Mass Spectrometry (LC–MS/MS) based untargeted metabolomics of the selected strains. Here, we report the isolation, taxonomical identification, cultivation and extraction of plaice-associated bacteria and fungi, as well as the results of their rapid metabolome and bioactivity profiling against pathogens causing human and fish diseases.

## 2. Results

### 2.1. Strain Isolation and Cultivation

In order to isolate a broad microbial diversity of plaice microbiota, three different agar media, namely Tryptic Soy medium (TSB12+5), modified Wickerham medium (WSP15) and Potato Dextrose medium (PDA), were used. WSP15 and PDA media are known to support mainly yeast and fungal growth [19,20]. TSB is a complex general-purpose medium for isolation and cultivation of a wide variety of bacteria and fungi. Additionally, we developed a fish medium (FM) to employ the Miniaturized Culture Chip (MCC) technique, which we previously employed for isolation of rare Gram-negative bacteria from Antarctic sediments [21,22]. FM was comprised of the gut content of fish as natural substrate and Baltic Sea water, as the aim was to mimic the natural microenvironment within fish gut, hence to improve cultivability of associated microorganisms. MCC contains thousands of microwells that allow an inoculation strategy where microbes grow on the upper side of the chip as segregated microcolonies, thereby can be isolated easily. As MCC was mostly used for sediment samples previously, the procedure was slightly adapted for this study: samples were diluted as described in the Material and Methods section and carefully pipetted on the fish medium. The MCC was then carefully put on top of the sample.

**Figure 1:**
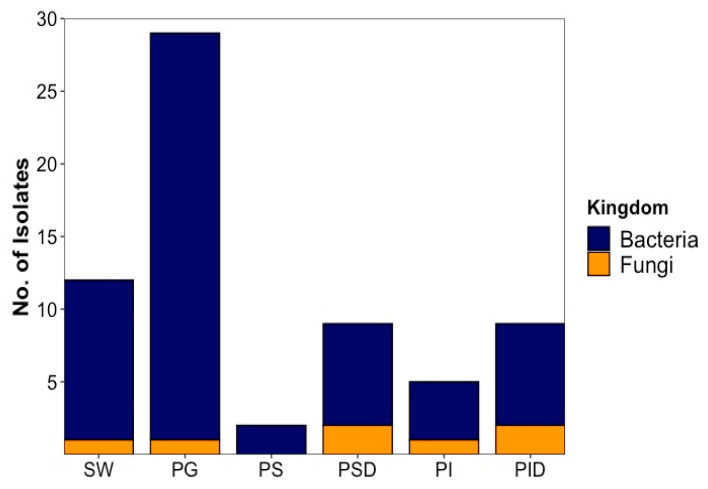
The origin and abundance of all isolated microorganisms by source. SW: Sea water, PG: Plaice Gill, PS: Plaice Stomach Epithelium, PSD: Plaice Stomach Digesta, PI: Plaice Intestine Epithelium, PID: Plaice Intestine Digesta.

In total, 66 microorganisms (59 bacterial and 7 fungal strains) were isolated. Of these, 54 were (48 bacterial and 6 fungal isolates) derived from fish, while 12 (11 bacterial and 1 fungal isolate) were retrieved from sea water reference (Figure 1). Isolate identification was performed by Sanger sequencing [23] of the PCR-amplified 16S rRNA gene for bacteria and ITS region for fungi followed by BLAST comparison to the NCBI nucleotide database (full database and type strains, Supplementary Table S1). As BLAST search frequently returned species of the same genus with identical similarity values, genus level was used as highest taxonomic level to ensure comparability. When species could be unambiguously identified, the species names are given in text and tables. Isolates were coded as PG: Plaice Gill, PI: Plaice Intestine Epithelium, PS: Plaice Stomach Epithelium, PID: Plaice Intestine Digesta, PSD: Plaice Stomach Digesta, SW: Sea water; MCC: Microchip (MCC) isolation, -B: Bacterium and -F: Fungus. Highest isolate numbers (29) were obtained from fish gills (28 bacterial and 1 fungal isolates), whereas 25 were GIT-derived (20 bacterial and 5 fungal isolates). Of the GIT-derived strains, digesta from stomach and intestines yielded equal numbers (9) of isolates (7 bacterial and 2 fungal isolates each). Only 2 bacterial isolates were obtained from the stomach epithelium, while the intestine epithelium afforded 5 isolates (4 bacteria and 1 fungus).

#### 2.1.1. Bacterial Isolates

**Figure 2:**
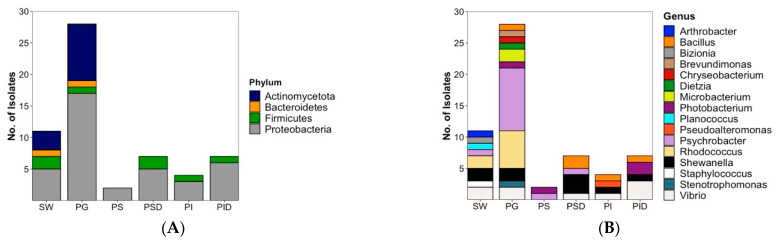
Overall diversity and distribution of 48 plaice-associated bacterial isolates in comparison to water reference (11 isolates) at (**A**) phylum level, (**B**) genus level. SW: Sea water, PG: Plaice Gill, PS: Plaice Stomach Epithelium, PSD: Plaice Stomach Digesta, PI: Plaice Intestine Epithelium, PID: Plaice Intestine Digesta.

Bacteria identified from cultivable plaice microbiota fall into 4 different phyla. With 33 out of 48 isolates, Proteobacteria (69%) represent the largest phylum, followed by Actinomycetota (9 isolates, 19%), Firmicutes (5 isolates, 10%) and Bacteroidetes (1 isolate, 2%). Members of the phyla Actinomycetota and Bacteroidetes were only isolated from the gill and sea water samples but not from the GIT. Proteobacteria and Firmicutes were the only isolated bacterial components of the GIT (Figure 2A).

Plaice-associated bacterial isolates were further classified into 11 families (Supplementary Figure S1). From the plaice GIT (stomach and intestine epithelia and digesta), 5 families (Pseudoalteromonadaceae, Vibrionaceae, Shewanellaceae, Moraxellaceae, Bacillaceae) were retrieved. The GIT was clearly dominated by the Gram-negative gammaproteobacterial families Vibrionaceae (8 isolates) and Shewanellaceae (5 isolates), followed by the Gram-positive Bacillaceae (order Bacillales, 4 isolates). Stomach and intestine epithelia shared the family Vibrionaceae, but other families obtained from this tissue differed (Moraxellaceae in stomach epithelium, Shewanellaceae, Pseudoalteromonadaceae and Bacillaceae in intestine epithelium). A similar trend was observed for digesta, which was clearly dominated by Vibrionaceae in the intestines, whereas Shewanellaceae was more prominent in the stomach. The most abundant gill dwellers, however, belonged to the gammaproteobacterial family Moraxellaceae (10) and the actinobacterial family Nocardiaceae (6, Supplementary Figure S1). Gill-associated microbiota also accounted for higher diversity (10 families) than gut-associated microbiota (5 families). Notably, Actinobacterial families Microbacteriaceae and Dietziaceae, the alphaproteobacterial family Caulobacteraceae, gammaproteobacterial family Lysobacteriaceae and Weeksellaceae belonging to the Flavobacteriia were only isolated from gills.

At genus level, the most diverse bacterial community was isolated from gills reflected by 11 different genera (Figure 2B). With 2 genera (*Psychrobacter* and *Photobacterium*), lowest diversity was observed for stomach epithelium. The 20 plaice GIT-associated bacterial isolates were dominated by gammaproteobacterial genera, namely 5 *Vibrio* sp. (3 from intestine digesta, 1 from stomach digesta and 1 from intestine epithelium), 5 *Shewanella* sp. (3 from stomach digesta, 1 from intestine epithelium and 1 from intestine digesta), 3 *Photobacterium* (2 from intestine digesta, 1 from stomach epithelium), and one *Pseudoalteromonas* sp. (from intestine epithelium). *Bacillus* represented the only nonproteobacterial genus in the gut with 4 isolates (2 from stomach digesta, 1 from intestine epithelium and 1 from intestine digesta).

In contrast, the 28 bacterial gill isolates were dominated by only two genera, namely *Psychrobacter* (10 isolates) and *Rhodococcus* (6 isolates), followed by 2 isolates each of *Shewanella, Vibrio* and *Microbacterium*, and 1 isolate each from *Bacillus*, *Brevundimonas*, *Chryseobacterium*, *Stenotrophomonas*, *Photobacterium* and *Dietzia* sp. (Figure 2B).

Inoculation of the seawater reference sample from the catch site yielded a reasonable diversity of isolates (Figure 2B) with a total number of 11 bacterial isolates belonging to 8 families (shown in Supplementary Figure S1): Vibrionaceae (2 strains), Shewanellaceae (2), Nocardiaceae (2), Moraxellaceae (1), Flavobacteriaceae (1), Micrococcaceae (1), Caryophanaceae (1) and Staphylococcaceae (1). Although several families were shared by the gut or gill-associated bacteria, 4 isolates from 4 different families, namely Flavobacteriaceae, Staphylococcaceae, Caryophanaceae and Micrococcaceae, were exclusive to sea water. At genus level, the 11 sea water-derived isolates were composed of 2 *Vibrio*, *Shewanella*, and *Rhodococcus* strains each, followed by 1 isolate each of the genera *Psychrobacter*, *Staphylococcus*, *Planococcus*, *Bizionia* and *Arthrobacter* (Figure 2B). The last 4 mentioned genera were exclusively retrieved from the sea water reference.

**Figure 3:**
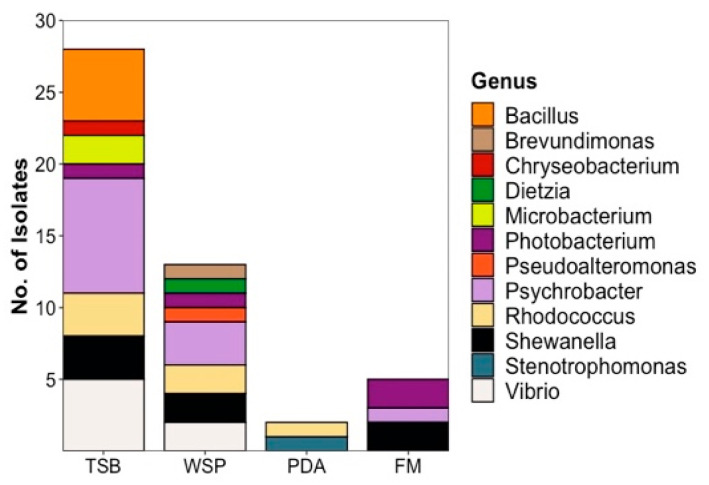
Diversity and distribution of plaice-derived bacteria recovered from different media at genus level. Seawater-derived isolates are shown in Supplementary Figure S3. TSB: Tryptic Soy medium, WSP: Wickerham medium, PDA: Potato Dextrose medium, FM: Fish medium.

The media used for cultivation of the microorganisms influenced the number of bacterial isolates recovered (Figure 3). Isolation efforts enabled recovery of the majority of the 48 plaice-associated isolates (28, corresponding to 58.5%) from the TSB12+5 medium. These isolates belong to 7 different families, namely Moraxellaceae (8, all *Psychrobacter* sp.), Vibrionaceae (6, 5 *Vibrio* sp., 1 *Photobacterium* sp.), Bacillaceae (5, all *Bacillus* sp.), Shewanellaceae (3, all *Shewanella* sp.), Nocardiaceae (3, all *Rhodococcus* sp.), Microbacteriaceae (2, both *Microbacterium* sp.) and Weeksellaceae (1, *Chryseobacterium* sp.) as shown in Supplementary Figure S2. WSP15 medium yielded lesser number of isolates (13, representing 27%) but—albeit partly different families than TSB—the same overall number (7). The families retrieved from WSP15 medium were Vibrionaceae (3, 2 *Vibrio* sp. and 1 *Photobacterium* sp.), Moraxellaceae (3, all *Psychrobacter* sp.), Nocardiaceae (2, both *Rhodococcus* sp.), Caulobacteraceae (*Brevundimonas mediterranea*), Dietziaceae (*Dietzia* sp.), Pseudoalteromonadaceae (*Pseudoalteromonas* sp.) and Shewanellaceae (2, *Shewanella* sp.). PDA medium, although originally applied for cultivation of fungi, yielded 2 bacterial isolates from 2 different families (4%, Nocardiaceae—*Rhodococcus* sp., Lysobacteraceae—*Stenotrophomonas* sp.). Only 5 (representing 10.5%) plaice-associated isolates from 3 families were retrieved from the FM medium, namely Shewanellaceae (2, both *Shewanella* sp.), Vibrionaceae (2, *Photobacterium* sp.) and Moraxellaceae (*Psychrobacter* sp., Supplementary Figures S2 and S3). As for the seawater isolates, the highest diversity was also obtained from TSB 12+5 medium (7 isolates from 7 different families (*Psychrobacter* sp., *Vibrio* sp., *Staphylococcus* sp., *Planococcus* sp., *Bizionia* sp., *Arthrobacter* sp. and *Shewanella* sp.) Two isolates were obtained from PDA (*Rhodococcus* sp.) and 1 bacterial isolate each from WSP (*Vibrio* sp.) and FM (*Shewanella* sp.) media (Supplementary Figure S3).

#### 2.1.2. Fungal Isolates

**Figure 4:**
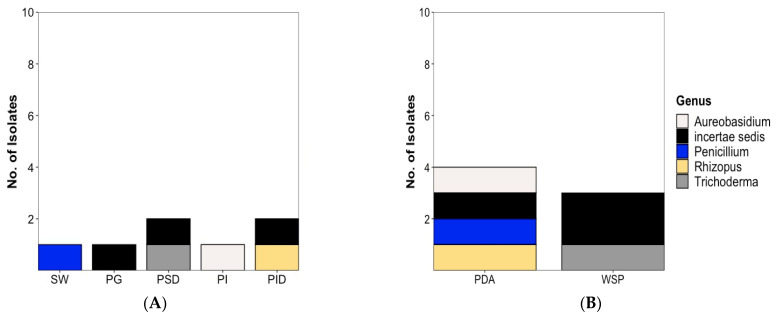
Diversity and distribution of fungal isolates (**A**) at genus level and (**B)** by isolation medium. Note: Figure 4 includes *Penicillium* sp. (colored in blue) isolated from the seawater reference sample. SW: Sea water, PG: Plaice Gill, PSD: Plaice Stomach Digesta, PI: Plaice Intestine Epithelium, PID: Plaice Intestine Digesta. No fungal isolate was obtained from Plaice Stomach Epithelium. PDA: Potato Dextrose medium, WSP: Wickerham medium.

The diversity of fungal strains was significantly lower and in total, only 7 fungal strains were isolated (6 from the plaice and 1 from the sea water sample). Except for one isolate, i.e., *Rhizopus* sp. Belonging to the phylum Mucoromycota, all fungal isolates are members of the phylum Ascomycota. Three fungal isolates could not be safely identified below class level and are shown as “incertae sedis” in Figure 4. A total of 5 isolates (Figure 4A) were associated to the plaice GIT, i.e., 2 each from stomach (*Trichoderma* sp. (PSD10-F), Sordariomycetes sp. Incertae sedis (PSD14-F)) and intestine digesta (*Rhizopus* sp. (PID1-F), Sordariomycetes sp. Incertae sedis (PID20-F)) and 1 from gut epithelium, *Aureobasidium pullulans* (PI9-F).

As displayed in Figure 4, only 1 fungal isolate each was obtained from gills (*Leotiomycetes* sp. Incertae sedis) and the seawater sample (*Penicillium* sp.). Four fungal strains (*A. pullulans, Rhizopus* sp., *Sordariomycetes* sp. Incertae sedis, and *Penicillium* sp. From water reference) were recovered from the PDA medium and 3 from the WSP15 medium (Figure 4B). Supplementary Table S2 shows the taxonomical identification of fungi isolated from plaice and the water sample to the highest possible taxonomic level.

### 2.2. Cultivation, Extraction and Bioactivity Assessments

From 66 total isolates, 54 were obtained from the plaice. Out of these 54 fish-associated isolates, a subset of 21 bacterial and 2 fungal strains was selected for cultivation. Selection of bacterial strains was based on several criteria: (i) only fish-associated microorganisms were selected, i.e., sea water isolates were removed, (ii) an almost equal distribution of selected isolates among the different fish body sites, i.e., 13 from gills, 10 from GIT and from the GIT 5 strains each from epithelium and digesta and (iii) phylogeny, i.e., isolates with different 16S rRNA gene sequences (e.g., four different gill-associated *Rhodococcus* sp. PG19-B, PG25-B, PG26-B and PG31-B), i.e., isolates having identical sequences were removed. From fungi, only the GIT epithelium (PI9-F: *A. pullulans*) and gill (PG36-F: Leotiomycetes sp.) strains were selected (no digesta strains). Table 1 shows the selected list of strains, their sources and bioactivities.

Bacteria were grown in both solid and liquid culture regimes using marine agar (MA, solid) and marine broth (MB, liquid). Similarly, the fungi were grown on potato dextrose agar (PDA) and in potato dextrose broth (PDB). These media were chosen because of our experience regarding their ability to sustain growth of a wide variety of marine bacteria and fungi, respectively. Consequently, all chosen strains showed good growth, except for a few strains, e.g., *Shewanella colwelliana* PSD4-B, *Shewanella aestuarii* PID2-B and *Photobacterium* sp. MCC-PID1-B, that showed poor or no growth in some media. The cultures were extracted with EtOAc. Crude extract yields ranged from 1.15 to 8.7 mg per duplicate of 10 agar plates and 2.75 to 36.9 mg per 500 mL broth with the highest extract amount being provided by the PDB liquid culture of fungal strain *A. pullulans* (PI9-F). On average, the extract yield obtained by liquid fermentation was three times higher (highest: 6.3-fold *Vibrio aestuarianus* PG12-B). The crude extracts were tested for their broad spectrum antimicrobial activity against a large panel containing (i) fish pathogens (*Lactococcus garvieae*, *Vibrio anguillarum*, *Vibrio ichthyoenteri*), human pathogens (ii) highly virulent, multidrug-resistant human bacterial panel (ESKAPE) pathogens, i.e., *Enterococcus faecium* (Efm), methillicin-resistant *Staphylococcus aureus* (MRSA), *Klebsiella pneumoniae*, *Acinetobacter baumannii, Pseudomonas aeruginosa* and *Escherichia coli* and (iii) fungal pathogens *Candida albicans* and *Cryptococcus neoformans*.

Of the selected 23 microorganisms, 13 (56.5%) showed activity in one or more bioassays at the initial test concentration of 100 µg/mL. The observed activities were limited to fish pathogens *V. ichthyoenteri* and *L. garvieae* and two Gram-positive human pathogenic bacteria, MRSA and *E. faecium*. No antifungal activity was observed with any of the extracts towards *C. albicans* or *C. neoformans*. Table 1 shows the IC_50_ values of the bioactive microbial extracts derived from both solid and liquid culture regimes, while Table S3 displays the bioactivity data of all 23 selected microbes. The majority (9) of the 13 active extracts derived from the gut, namely the epithelium (4 bacteria, PI1-B, PI2-B, PI8-B and PS3-B and 1 fungus PI9-F) or digesta (4 bacteria, PID21-B, MCC-PID1-B, PSD4-B and PID2-B). Only four bacteria sourced from the plaice gills (PG1-B, PG10-B, PG11-B, and PG12-B) exerted activity, showing the higher potential of gut-associated microorganisms for applications.

Starting with bacteria, four bacterial strains (*Chryseobacterium carnis* PG10-B, *Microbacterium* sp. PG11-B, *V. aestuarianus* PG12-B and *Bacillus* sp. PID21-B) inhibited the growth of the Gram-negative fish pathogen *V. ichthyoenteri* (Table 1). The gill-derived *C. carnis* (PG10-B) moderately inhibited this pathogen only when cultured in solid (MA) medium (IC_50_ 18.6 µg/mL). The opposite held true for the gill-derived *Microbacterium* sp. (PG11-B) that showed high inhibition (IC_50_ 5.7 µg/mL) only upon cultivation in marine broth (liquid regime). Both the liquid and solid culture extracts of the gill-derived *V. aestuarianus* (PG12-B) and gut-derived *Bacillus* sp. (PID21-B) displayed activity against this emerging fish pathogen. Notably, their solid culture extracts were 3 to 6 times more active (Table 1). Overall, the highest potency against *V. ichthyoenteri* was exerted by *Bacilllus* sp. (PID21-B, MA medium, IC_50_ 4.7 µg/mL) and *Microbacterium* sp. (PG11-B, MB medium, IC_50_ 5.7 µg/mL). Although only few bacteria showed activity against this pathogen, the solid marine agar medium extracts tended to exert higher activity than the liquid marine broth extracts (Table 1).

Moderate activities were demonstrated by 7 bacterial strains (*Shewanella baltica* PG1-B, *V. aestuarianus* PG12-B, *Bacillus* sp. PI1-B, *Vibrio* sp. PI2-B, *Pseudoalteromonas* sp. PI8-B, *Photobacterium* sp. MCC-PID1-B, *Shewanella colwelliana* PSD4-B) against *L. garvieae* (Table 1). The highest inhibition was displayed by the solid culture extracts of gut-derived bacteria *Vibrio* sp. PI2-B and *Bacillus* sp. PI1-B with IC_50_ values of 14.1 and 14.7 µg/mL. The MB (liquid) culture extract of the latter (*Bacillus* sp. PI1-B) was also active but with almost 4 times lower potency (IC_50_ 54.2 µg/mL). The MA and MB-derived extracts of *Pseudoalteromonas* sp. PI8-B were almost equally active (IC_50_ values 28.3 and 36.6 µg/mL, respectively). Gill-derived bacteria *V. aestuarianus* (MA, PG12-B) and *S. baltica* (MB, PG1-B) and stomach-digesta originated *S. colwelliana* (MA, PSD4-B) extracts showed mild activities (IC_50_ values 27.8, 41.7 and 25.4 µg/mL, respectively). Finally, the intestine-digesta-derived bacterium *Photobacterium* sp. MCC-PID1-B was moderately active (MA medium, IC_50_ value 40.2 µg/mL). Similar to the results observed against *V. ichthyoenteri*, generally, the solid media extracts were more active than the extracts of the liquid cultures. The third fish pathogen *V. anguillarum* was not affected by any of the bacterial extracts, even at the highest test concentration 100 µg/mL.

As for the human pathogens, only MRSA and *E. faecium*, two Gram-positive bacteria from the ESKAPE panel were susceptible to plaice-associated bacterial extracts, while the gram-negative bacterial pathogens in the ESKAPE panel were not inhibited by any of the extracts. Overall, 12 bacterial strains were active against MRSA with IC_50_ values ranging from 5.9 to 61.0 µg/mL. We observed a general trend that the liquid fermentation (MB) extracts were more active, and indeed, the highest potency was observed with the MB extracts of three gut-derived bacteria, namely *Bacillus sp.* PID21-B (IC_50_ 5.9 µg/mL), *S. aestuarii* PID2-B (6.8 µg/mL) and *Pseudoalteromonas* sp. PI8-B (8.1 µg/mL). The remaining extracts had IC_50_ values ranging from 10.2 to 45.9 µg/mL (Table 1).

As shown in Table 1, the majority (12) of bacterial strains also showed activity against *E. faecium*. The general trend, i.e., the exhibition of more prominent antimicrobial activity by the liquid MB extracts held also true for this pathogen and the extracts that inhibited the growth of MRSA generally showed efficacy against *E. faecium.* For example, the liquid medium extracts of *Pseudoalteromonas* sp. PI8-B were equally active against both human pathogens (Table 1). The highest potency was observed with the liquid extracts of five gram-negative bacteria associated with plaice. One of them derived from the intestine digesta, *S. aestuarii* PID2-B (IC_50_ 4.9 µg/mL); three were epithelium-associated, i.e., *Psychrobacter sp.* PS3-B (IC_50_ 7.5 µg/mL), *Pseudoalteromonas* sp. PI8-B (IC_50_ 8.2 µg/mL) and *Vibrio* sp. PI2-B (IC_50_ 9.0 µg/mL); while the fifth was a gill-associated bacterium, *S. baltica* PG1-B (IC_50_ value 8.8 µg/mL). As displayed in Table 1, the remaining extracts showed activities ranging from notable to low with IC_50_ values ranging from 11.6 to 86.6 µg/mL.

As for the fungi, only the intestine-epithelium-derived fungus *A. pullulans* PI9-F was active. As displayed in Table 1, its PDA (solid) extract was moderately active against the fish pathogen *L. garvieae*, MRSA and *E. faecium* with IC_50_ values 22.6, 40.8 and 30.7 µg/mL, respectively. The liquid PDB extract, however, strongly inhibited *E. faecium* (IC_50_ 3.3 µg/mL) and MRSA (IC_50_ 8.9 µg/mL). The former activity against *E. faecium* represents overall the highest activity obtained with any plaice-associated microorganisms in this study.

**Figure 5:**
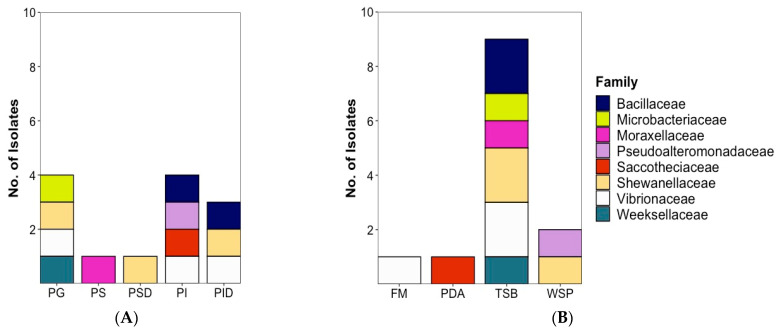
Taxonomic distribution of 13 bioactive microorganisms at family level according to (**A**) isolation source and (**B**) isolation medium. Note that the Figure 5 contains also the bioactive fungal isolate (*A. pullulans* PI9-F, colored in red). PG: Plaice Gill, PS: Plaice Stomach Epithelium, PSD: Plaice Stomach Digesta, PI: Plaice Intestine Epithelium, PID: Plaice Intestine Digesta. FM: Fish medium, PDA: Potato Dextrose medium, TSB: Tryptic Soy medium, WSP: Wickerham medium.

When considering the taxonomic affiliation of the bioactive plaice-associated microorganisms, we observe an interesting trend. As shown in Figure 5 (at phylum level in Supplementary Figure S4), the majority of the active isolates (9 out of 13) are Gram-negative bacteria; 8 being Proteobacteria belonging to families Vibrionaceae (3, *V. aestuarianus* PG12-B, *Vibrio* sp. PI2-B, *Photobacterium* MCC-PID1-B), Shewanellaceae (3, *S. baltica* PG1-B, *S. colwelliana* PSD4-B, *S. aestuarii* PID2-B), Pseudoalteromonadaceae (1, *Pseudoalteromonas* sp. PI8-B) and Moraxellaceae (1, *Psychrobacter* sp. PS3-B), and one belonging to the Flavobacteriia family Weeksellaceae (*C. carnis* PG10-B). Gram-positive bacteria are represented by two members of the phylum Firmicutes (*Bacillus* sp. PID21-B and PI1-B) and 1 actinobacterium (*Microbacterium* sp. PG11-B, phylum Actinomycetota). The only active fungal strain *A. pullulans* PI9-F belongs to the phylum Ascomycota, family Saccotheciaceae (Figure 5A). As for the isolation medium, all bioactive isolates but 4 were isolated on TSB medium (Figure 5B).

### 2.3. Molecular Network-Based Untargeted Metabolomics

All 13 bioactive isolates (12 bacterial strains and one fungal strain) were selected for an in-depth UPLC–QToF–MS/MS-based untargeted metabolomics. Feature-based molecular network tool (FB MN, [24]) served as a foundation for evaluation of the overall metabolome profile of the extracts. GNPS-based automated dereplication was combined with manual dereplication to identify the widest variety of known and potentially undescribed molecules in the crude extracts. When necessary, the dereplication of the bacterial extracts was assisted by their phylogeny.

**Figure 6:**
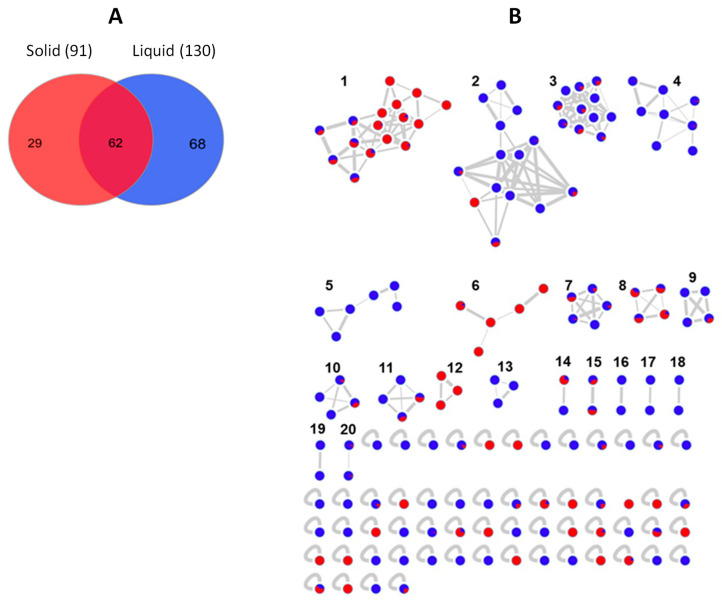
(**A**) Venn diagram displaying chemical diversity of the bacterial strains based on the culture regime. (**B**) Global molecular network of all bioactive bacterial extracts derived from solid (red) and liquid (blue) cultures. Clusters are numbered consecutively (1 to 20), edge widths correspond to the respective cosine score. The following clusters were putatively annotated. Cluster 2: Phosphatidylethanolamines, Clusters 3 and 4: Lipids, Cluster 6: Lipoamides, Cluster 8: Isocoumarins, Cluster 12: Bile acids. The remaining clusters have returned no hits in database search.

The global bacterial molecular network contained overall 159 nodes and 20 clusters (Figure 6B). Of these nodes, 29 (18%) were solely expressed in solid media cultures, while 68 (43%) were exclusive to the liquid cultures. The remaining 62 nodes (39%) were shared by the extracts of both liquid and solid media (Figure 6A). Several small molecular families, i.e., clusters 5, 13, 16–19 were only detected in the liquid medium extracts, while the bile acid cluster 12 was exclusive to the solid (MA) extracts.

Only 6 clusters out of 20 turned one or more compounds that could be annotated by GNPS or manual dereplication process using various public or commercial databases. Overall, the automated dereplication of the bacterial raw data by GNPS workflow led to the putative identification of 14 compounds based on their spectral similarities with natural products from online databases. Most of these compounds represent primary metabolites such as fatty acids, phosphatidylethanolamines or amino acids. Some of those identified metabolites, e.g., phosphatidylethanolamines are constituents of bacterial membranes [25]. Overall, 64% of the nodes were exclusive to one bacterial strain, i.e., *Bacillus* sp. (PID21-B) while the remaining nodes were shared by several different bacteria. Of particular interest were those nodes unique to individual strains or shared by taxonomically closely related species, as they are presumably secondary metabolites. Nevertheless, all shared nodes were considered in the annotation workflow and given when significant. Here, we discuss in more detail the bioactive and chemically richest strains, i.e., bacterial strain *Bacillus* sp. (PID21-B) and the only bioactive fungus *A. pullulans* (PI9-F). A detailed dereplication information on each bioactive strain is enclosed in Tables S4–S15.

**Figure 7:**
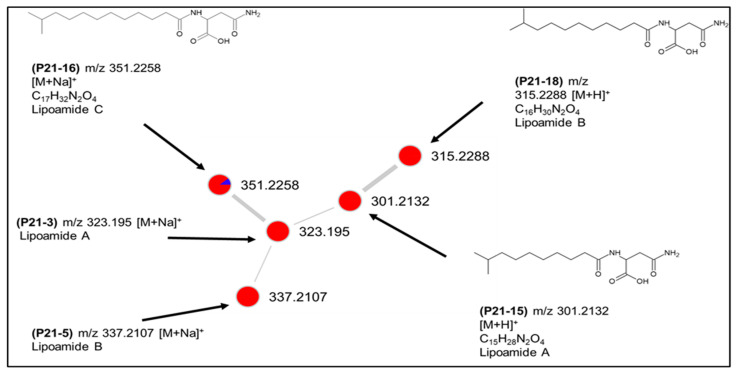
Putative lipoamide cluster (6) produced by *Bacillus* sp. PID21-B.

As mentioned above, *Bacillus* sp. PID21-B had the greatest chemical diversity of all bacterial strains having 22 unique nodes in the global network. It also accounted for the highest number of putatively annotated metabolites in one strain (Table S4). Furthermore, two clusters in the global molecular network (Figure 6B), namely clusters 6 and 8, were exclusive to this species. Cluster 6 was assigned to lipoamides A–C and their sodium adducts (shown as P21-3, P21-5, P21-15, P21-16, P21-18, Figure 7). They are simple, linear aminolipids originally isolated from the marine sediment bacterium *Bacillus pumilus.* Lipoamide A shows weak antibacterial activity against *S. aureus* and *P. aeruginosa* [26]. All nodes, except for lipoamide C (P21-16 *m/z* 351.2258 [M + Na]^+^), were solely detected in the solid culture, indicating the major influence of culture regime on chemical diversity (Figure 7).

**Figure 8:**
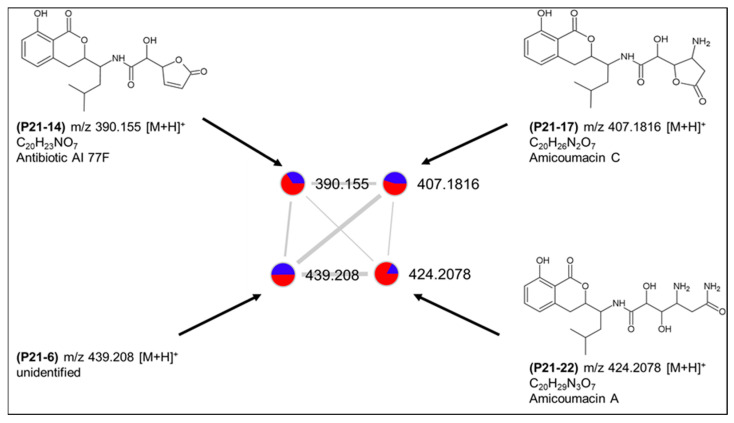
Putative dihydroisocoumarin cluster (8) produced by *Bacillus* sp. PID21-B.

The second cluster (cluster 8) comprised four nodes (P21-6, P21-14, P21-17 and P21-22) and was putatively annotated as amicoumacins A, C and antibiotic AI77 F (Figure 8), all originally reported also from *B. pumilus* [27,28]. These molecules belong to the bacterial dihydroisocoumarins that have been obtained from different *Bacillus* and *Nocardia* species with a broad spectrum of bioactivities [29]. The fourth ion (P21-6) in the cluster with *m/z* 439.208 [M + H]^+^ could not be linked to any described structure and may represent a new derivative. All four peaks were detected in both solid and liquid cultures and, therefore, can be considered to be at least partially responsible for the observed bioactivities of this species. As shown in Table S4, a few more, individual compounds that belong to other natural product classes, such as polyketides, macrolides or terpenoids were also putatively annotated.

The second *Bacillus* sp. (PI1-B) accounted for 5 unique nodes only one of which (PI1-1, *m/z* 417.1489, C_15_H_26_N_2_O_10_ [M + Na]^+^) was annotated as enkastine 2 [30], a glycopeptide isolated from *Streptomyces albus* with antiendopeptidase activity (Supplementary Table S5). The other Gram-positive actinobacterium *Microbacterium* sp. (PG11-B) only accounted for one (unique) peak (PG11-1) detected at *m/z* 716.5224 that could not be annotated (Table S6).

Moving to the Gram-negative associated bacteria, *C. carnis* (PG10-B) had a large number of nodes (14) in the MN. Molecular clusters 9 and 15, which were expressed almost solely in liquid culture (Figure 6) were exclusive to *C. carnis* extract. They returned no hits in database searches, indicating their potential novelty. Only few compounds from this species putatively annotated, including two singletons (i) PG10-4 (C_18_H_38_O_3_) as 3-hydroxy-16-methylheptadecanoic acid originally derived from *Bacillus* sp. and (ii) PG10-5 *m/z* 490.1445 [M + H]^+^ as polyoxin O, a nucleoside antibiotic [31] (Table S7).

From the gill-derived *S. baltica* (PG1-B), only two steroidal bile acids were dereplicated by GNPS (Table S8). The gut digesta derived *S. colwelliana* PSD4-B and *S. aestuarii* PID2-B had growth problems in the liquid or solid media, respectively. The automated dereplication on available liquid culture extract of *S. aestuarii* (PID2-B, Table S9) annotated a linoleic acid derivative 10E,Z12-CLA while another compound was manually assigned to an α-pyrone, violapyrone C, an antibacterial agent isolated from a *Streptomyces* sp. [32]. Two additional compounds were annotated putatively as the antifungal macrolide antibiotic 21-deoxybafilomycin A2 and 35-amino-3β-methylbacteriohop-11-ene-30,31,32,33,34-pentol that were described from a methanotrophic gammaproteobacterium *Methylocaldum* sp. [33]. No annotation was possible for the constituents of *S. colwelliana* (PSD4-B, Table S10).

The gammaprotobacterium *Psychrobacter* sp. (PS3-B) only accounted for a few unique peaks in the network. Three of these unique peaks clustered together (cluster 12 in global network, Figure 6) and were automatically annotated as different adducts of the same bile acid (Table S11). Although literature on chemistry of this genus is extremely scarce, the finding corresponds to a study that describes a *Psychrobacter* species as producer of novel bile acid derivatives [34]. The other two nodes were singletons and could not be annotated.

The extracts of *V. aestuarianus* PG12-B and *Vibrio* sp. PI2-B contained respectively twelve and seven unique nodes each. The cluster 5 from the global molecular network (Figure 6) comprising 6 nodes was exclusive to these two *Vibrio* sp. We were able to assign two compounds uniquely produced by *V. aestuarianus* as polyketides, ethyl tumonoate A that shows anti-inflammatory activity [35] and 4-hydroxy-5-(hydroxymethyl)-3-(14-methylhexadecanoyl)-2(5H)-furanone, a protease inhibitor [36]. Cluster 13 containing 3 nodes was unique to *V. aestuarianus*, yet none of the nodes could be annotated. The antimalarial alkaloid marinacarboline C (PI2-6, C_22_H_19_N_3_O_2_) [37] was unique to *Vibrio* sp. PI2-B *(*Tables S12 and S13). The third Vibrionaceae bacterium *Photobacterium* sp. (MCC-PID1-B) had no interesting chemistry and did not produce any ion exclusive to it. The crude extract of the final gram-negative protobacterium *Pseudoalteromonas* sp. (PI8-B) accounted for four unique nodes. Only one of these led to a database hit, albocycline B (C_18_H_30_O_5_), a macrolide originally isolated from *Streptomyces* sp. with weak antimicrobial activity against Gram-positive bacteria [38]. The other nodes could not be matched to any known compounds (Table S14).

**Figure 9:**
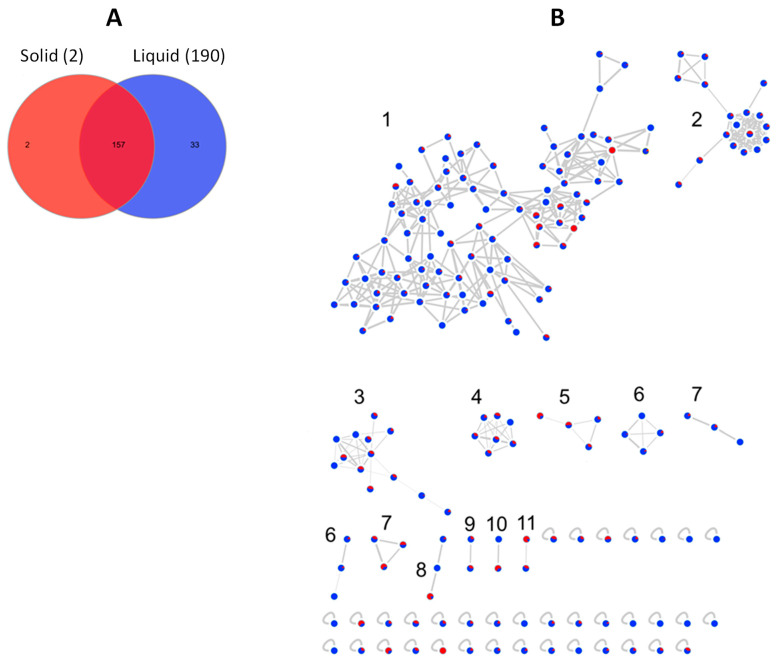
Chemical profile of *A. pullulans* PI9-F. (**A**) Venn diagram. (**B**) Feature-based molecular network showing the number of ions detected in solid (red) and liquid (blue) cultures. The clusters in the network are numbered consecutively, edge width represents cosine score. Clusters 1, 2 and 7 were annotated as 3,5-dihydroxydecanoic acid derivatives.

The molecular network of the fungus *A. pullulans* (PI9-F) consisted of 192 nodes within 11 clusters (Figure 9B). The majority (157) of the nodes were commonly found in both solid and liquid culture extracts. Nevertheless, 33 nodes were unique to the liquid media, while only two were solely present in the solid medium (Figure 9A). This is in line with the general trend observed with bacterial isolates that displayed a higher chemical diversity in liquid culture medium.

The molecular network of *A. pullulans* PI9-F was dominated by a large molecular family (cluster 1) containing 91 nodes and accounting for 58% of the total node numbers. As shown in Figure 9B, the majority of the nodes within cluster 1 were components of liquid media extracts. From cluster 1, we were able to annotate automatically a few nodes as complex derivatives of the 3,5-dihydroxydecanoic acid, a fatty acid commonly produced by this fungus [39]. Among them were 3-hydroxy-1-oxo-1-(2,3,4,5,6-pentahydroxyhexoxy)decan-5-yl] 3,5-dihydroxydecanoate (*m/z* 577.3199 [M + Na]^+^,), 5-[5-[5-(3,5-dihydroxydecanoylox)-3-hydroxydecanoyl]oxy-3-hydroxydecanoyl]oxy-3-hdroxy-decanoic acid (*m/z* 745.5097 [M − H_2_O + H]^+^). Additional manual dereplication efforts permitted several more annotations, including the node *m/z* 399.1947, [M + Na]^+^ as the antibacterial fatty acid 3,6,8,11-tetrahydroxy-16,17-dimethyloctadecanoic acid [40]. Another compound with *m/z* 388.2096 [M + Na]^+^ was putatively annotated as 7-O-(*N*,*N*-dimethylaminoacetyl) brefeldin A. Brefeldin A is a macrocyclic lactone that has been isolated from several *Penicillium* sp. and described as an antiviral compound [41]. Supplementary Table S15 shows all other putatively annotated molecules.

## 3. Discussion

Fish represent a diverse, large vertebrate group playing important roles in marine food webs, as ecosystem engineers, vectors for microorganisms [42] and protein source for human nutrition. However, the microbial ecology of many fishes has remained underexplored so far, although beneficial contributions of microbial assemblages to their host are known [43]. Fish harbor a complex and dynamic microbiota on their mucosal surfaces and intestines [44] driven by a complex interlinkage of external, physiological, dietary and evolutionary factors [43]. Microbial community composition can be actively selected and enriched by the fish [45]. Production of antimicrobial compounds by commensal microbes, which has been suggested for application as a less harmful alternative to traditional antibiotics in aquaculture [46] is only one way of contribution of microbiota to fish health. This is of particular economical relevance, because increasing resistances have a become a major threat to the fastest growing food production sector of the world [47]. The potential of fish associated microorganisms for drug discovery against human diseases is also being realized [48,49,50,51].

The fish gill is the primary site for gas and waste exchange [52,53], mucosal immune interactions, osmoregulation and detoxification [54], hence gill-associated microbiota are important for overall fish physiology and health. Gills act as the first line of defense against environmental pathogens [55,56]. The mucus coating the gills hosts an array of indigenous microorganisms, particularly bacteria [57,58,59]. In our study, gill-associated bacteria yielded higher number of isolates and diversity (28 bacteria, 10 families) compared to the gut (20 isolates, 5 families). Higher bacterial diversity in fish external mucosa (skin and gills) was already reported by Lowrey et al. [60] in a culture-independent study using 16S rRNA pyrosequencing. External surfaces act as interface between fish and surrounding water and therefore may be influenced by environmental factors as shown for Atlantic salmon [59], while the gut provides a more protected and stable environment hosting more specialized microbial communities. Another culture-independent study on bacterial communities associated with gill mucosa of four butterflyfish species showed a high Shannon diversity in all species [61]. Our isolates from plaice gills were dominated by the members of Gammaproteobacteria (families Moraxellaceae, Vibrionaceae, and Shewanellaceae), which is in alignment with other reports on marine fish gills [42,57,59]. The next abundant phylum was Actinobacteria (Microbacteriaceae, Nocardiaceae, and Dietziaceae) with dominance of *Rhodococcus* sp. (Nocardiaceae). *Rhodococcus* sp is an ammonia-oxidizing bacterium with simultaneous aerobic denitrification abilities [62], is extremely oligotrophic and CO_2_-dependent [63], hence is a successful gill colonizer. It has been tested as probiotic [64] and reported to show bacteriocinogenic properties against human and fish pathogens [65]. However, our selected *Rhodococcus* sp. isolates (PG19-B, PG25-B, PG26-B, and PG31-B) did not show any notable bioactivity (Supplementary Table S3) and were therefore disregarded for further chemical analysis.

Gut microbiota of marine fish also has significant impact on fish health by regulating complex and relatively stable microbial–microbial and host–microbial relationships. It is crucial for the development and innate immunity of the host and nutrient metabolism [66]. It arises from the egg epiflora during fish larvae hatching [67]. However, upon start of active feeding the fish intestinal microflora will be primarily feed-derived [68,69,70] and the microbiome of an adult fish is a diverse assemblage of microbial taxa [57]. Although partly gut microbiota of adult fish was found to cluster with microbiota from their surrounding environment, many species were found to be GIT-specific [71], which further stipulates maintenance of key microbial species with specialized metabolic abilities. Based on their competitive ability for adhering to mucus and host physiological pressure, fish gut microbiota falls into two categories of autochthonous (epithelium associated) and allochthonous (food associated) organisms. Autochthonous microbiota is considered as a fingerprint for fish species [57,72,73]. In this study, we divided the gut microbiota of the Baltic plaice into these major groups (intestine and stomach epithelium and digesta) to be able to more precisely compare their chemical profile and diversity. Since the intestine was washed three times with sterile 1.5% saline water prior to sampling and plating, we conclude that 7 microorganisms (6 bacteria, 1 fungus) isolated from the GIT epithelia (2 from stomach, 5 from intestine) belong to the autochthonous category. Moreover, we defined 18 allochthonous microorganisms (14 bacteria, 4 fungi) associated with the digesta samples (7 bacteria, 2 fungi from stomach and intestines each). The six epithelium-associated bacterial isolates were clearly dominated by Proteobacteria (class: Gammaproteobacteria, 5 isolates in 4 different families: Moraxellaceae, Vibrionaceae, Shewanellaceae, Pseudoaltermonadaceae). Bacteria in digesta were more abundant but slightly less diverse with 11 Proteobacteria belonging to 3 families (Shewanellaceae, Vibrionaceae, Moraxellaceae) and 3 Firmicutes (all Bacillaceae). Proteobacteria, in addition to Bacteroidetes and Firmicutes, reportedly comprise 90% of the fish intestinal microbiota of the different species studied thus far [74,75]. This is supported by studies based on culture-independent approaches as performed by Estruch et al. [76]. Our data confirm these findings, as Proteobacteria comprised the majority (64.5%) of plaice (gill and gut)-associated microbiota. The plaice GIT was observed to harbor a large number of Vibrionaceae (*Vibrio* and *Photobacterium* sp.). The high abundance of *Vibrio* sp. may on the one hand indicate a high pathogen load of the sampled plaice specimens. Some *Vibrio* sp. is known to be pathogenic for plaice, e.g., *V. anguillarum* and *V. splendidus* have been associated to plaice mortality [77]. A second possibility are feed-associated *Vibrio* sp. Shellfish is, besides polychaetes and crustaceans, the main diet of plaice. Increased water temperatures have led to increased prevalence of *V. rotiferianus*, *V. jasicida* and *V. parahaemolyticus* at shellfish aquaculture sites [78]. Elevated *Vibrio* loads in shellfish may also give rise to elevated *Vibrio* numbers in the GIT of their predators, among them the plaice. On the other hand, *Vibrio* sp., and *Shewanella* sp. (together with *Aeromonas* sp.) reportedly play a pivotal role in immune stimulatory effects in fish, i.e., activation of neutrophils in response to infection or injury as shown in zebrafish [79], which also might be true for plaice. These findings underline that especially gut microbiota and their multispecies interactions are highly interesting for further microbial and metabolomic analysis.

In our culture-based study, isolation efforts from plaice yielded higher abundance and diversity for bacteria than for fungi. The majority of published reports on natural products from fungal associates of marine fish are from Capon group, who focus on the cultivable mycobiota of the warm-water mugil mullet fish (*Mugil cephalus*). Among their library of >500 fungal isolates, genera found generally in the marine realm are reported such as *Trichoderma, Scopulariopsis* and *Aspergillus* sp. [80] but also rather rarely isolated taxa such as *Beauveria*, *Evlachovaea*, *Metarhizium*, *Spiromastix*, *Chrysosporium* [51,81,82,83]. To our knowledge, all so far published fish associated fungi belong to the Ascomycota, whereas we also have isolated a Mucoromycota representative from plaice. Taxonomic affiliation of fungi beyond class level based on ITS sequences is often challenging, but most reported fungi are affiliated to Eurotiomycetes and Sordariomycetes. From our 7 fungal isolates, also 3 were affiliated to Sordariomycetes and one to Eurotiomycetes. We have isolated also a bioactive member of the class Dothideomycetes (*A. pullulans* PI9-F) and the rarely obtained Leotiomycetes, which showed however no bioactivity in our activity assays.

Fish intestine can harbor 10^7^ to 10^11^ bacteria/g of intestinal digesta, with aerobes and facultative anaerobes being more abundant than obligate anaerobes [84]. This increases their cultivability in comparison to mammal’s gut microbiota. Fish gills are reported to host a much lesser richness with 10^2^ to 10^6^ bacteria/g wet weight [85]. Despite this, the fish microbiome has been underexplored for drug discovery studies. Instead, soft-bodied, sedentary marine invertebrates and their microbial symbionts have long been the mainstay for the discovery of novel natural products so far [48]. Nonetheless there is an emerging interest in the last decade into fish associated bacteria and fungi, and wide variety of secondary metabolites with diverse bioactivities are being reported [48,49,50,51].

Plaice-associated isolates with activity against fish and/or human pathogenic microbes were derived from 4 bacterial phyla (Proteobacteria, Bacteroidetes, Firmicutes, Actinomycetota) and one fungal phylum (Ascomycota, Supplementary Figure S4). Interestingly, 61% of the active isolates were affiliated to families Pseudoalteromonadaceae, Shewanellaceae, Vibrionaceae, Moraxellaceae in the phylum Proteobacteria, class Gammaproteobacteria. From those, Shewanellaceae and Vibrionaceae families accounted for three active strains each. Notably, extracts of all three members of the Gram-negative genus *Shewanella* only displayed activity against gram-positive organisms, which may again be an indication for their important roles in the fish immune defense. The families Weeksellaceae (phylum Bacteroidetes) and Microbacteriaceae (phylum Actinobacteria) accounted for one bioactive isolate each and the phylum Firmicutes (Bacillaceae) was represented by 2 bioactive isolates. The fact that most of the bioactive strains are gram-negative bacteria in the present study is intriguing, because gram-positive Actinobacteria represent the main focus of the current microbial biodiscovery studies. Being source of prominent anticancer natural products such as didemnin B and bryostatins, Gram-negative marine bacteria are also being recognized as a prolific source of small molecules with strong biological activities [86,87,88]. The present study lends further support to their untapped potential and encourages systematic chemical studies on their constituents. Interestingly, five bacteria *C. carnis* (PG10-B), *Microbacterium* sp. (PG11-B), *V. aestuarianus* (PG12-B), *Bacillus* sp. (PID21-B) and the fungal strain *A. pullulans* (PI9-F) displayed activity against both Gram-positive and Gram-negative test organisms. The only bioactive fungal strain *A. pullulans* is a member of the family Saccotheciaceae (class Dothideomycetes, phylum Ascomycota). Phylum Ascomycota is well known as a rich source of complex and highly active natural products [89,90]. The solvent extracts of *A. pullulans* displayed bioactivity against the gram-positive pathogens *E. faecium*, MRSA and the fish pathogen *L. garviae*, when it was cultured on agar-based medium. Although no activity against Gram-positive test organisms was observed, fungi are historically prolific sources of antibiotics, and the liquid culture extract of *A. pullulans* exerted overall the lowest IC_50_ value, i.e., the highest antimicrobial potency in our study, warranting in-depth investigations in future.

Overall, the gut-derived bacteria showed higher and broader activity than the gill-associated bacteria. The only bioactive fungal strain *A. pullulans* derived from the gut of plaice, showed notable bioactivity. Altogether 90% of the selected gut-derived microorganisms were active, while only 31% gill-associated microbes studied herein showed bioactivity. Some researchers regard gut microbiota as “extra organ”, due to its crucial and multiple functions in the intestinal development and physiology, i.e., digestive processes, metabolism, gut motility, hormone release, intestinal barrier function, immune defense, and feeding behavior [91]. All these functions are related to chemical interactions between the microbiota and microbiome/host which are mostly achieved via small molecules (i.e., short chain fatty acids, hormones, neurotransmitters). Due to these important functions of gut microbiota in host homeostasis and health, it is explainable that a higher proportion of gut isolates than gill isolates showed bioactivity in our experiments.

As expected, the culture regime has also played a role in the overall activity profile, potency, as well as metabolome composition of the microbes. An interesting trend observed was that the solid media extracts exerted better potential against fish pathogens *V. ichthyoenteri* and *L. garvieae*, while liquid media extracts displayed higher potency against the human pathogens MRSA and *E. faecium*. Few strains (e.g., *Bacillus* sp. PI1-B, *Pseudoalteromonas* sp. PI8-B) showed activity when cultured in either regime. It should be mentioned that some strains did not grow in solid or liquid media, hence their activity could not be investigated in detail. The obtained biomass, hence yield of microbial extracts was considerably higher when liquid MB medium was used. Notably, the bioactive fungus *A. pullulans* (PI9-F) showed overall the highest yields when cultured in both solid (8.7 mg) and liquid media (36.9 mg). Unfortunately, application of MCC and fish medium did not enhance isolate diversity, all genera recovered with this technique (*Shewanella, Psychrobacter, Photobacterium*) were also retrieved using the classical isolation approach. As this technique has already proved its potential with application in marine sediment samples [21,22], further adaptation of the technique is necessary.

The untargeted metabolomics approach using Feature-based Molecular Networking (FBMN) indicated the chemical diversity of plaice associated microbiota. The liquid medium extracts did not only exhibit better activity profile (only human pathogens though); they also displayed a much richer chemical diversity. Overall, a large portion of metabolites (nodes) observed in the global molecular network of bacteria derived solely from one species, *Bacillus* sp. (PID21-B) while that of the fungus *A. pullulans* consisted of 192 nodes. Although many nodes belong to a lipid class, the chemical diversity of this gut derived fungus was remarkable. In comparison to fungi, bacterial extracts were less rich in chemistry, however we were still able to annotate some antimicrobial or otherwise bioactive microbial metabolites from their extracts that may be responsible or involved in the observed antimicrobial activity of the extracts. A number of molecular clusters did not return any hits in extended database searches, indicating them to potentially belong to so far undescribed molecular families. An extended One-Strain-MAny-Compounds (OSMAC) approach using different culture media and other varied culture conditions will certainly increase the chemical space of bacteria.

In conclusion, the gills and the gut of the economically important flatfish *Pleuronectes platessa* represent a unique niche for bacteria and fungi as source of potential antimicrobials useful against human and aquaculture-associated pathogens. Several isolates warrant large-scale cultivation studies followed by compound isolation and purification efforts.

## 4. Materials and Methods

### 4.1. General Procedures

Tandem mass (MS/MS) spectrometry data were recorded on a Waters Xevo G2-XS QToF Mass Spectrometer (Waters^®^, Milford, MA, USA) connected to a Waters Acquity I-Class UPLC system (Waters^®^, Milford, MA, USA) in positive mode. The organic solvent used for MS/MS analysis was ULC/MS grade. An in-house Arium^®^ Water Purification System (Sartorius, Goettingen, Germany) was used for the preparation of milli Q water. Potato extract and dextrose used for fungal cultivation were purchased from Sigma-Aldrich (Schnelldorf, Germany) and Merck (Darmstadt, Germany), respectively. Agar was purchased from Applichem (Darmstadt, Germany).

### 4.2. Sampling and Dissections

Five mature specimens of *Pleuronectes platessa* were caught in Kiel Bay in May 2019 and brought to the laboratory in a cool box immediately. Seawater taken at the site of catch served as reference sample. Dissection and microorganism isolation studies were conducted under sterile conditions on the same day.

For gill preparation, all four gill arches were removed with sterile surgical blades and placed into a sterile petri dish and washed in 1.5% sterile saline to remove transient bacteria and potential contaminations. Afterward, the gill tissue was homogenized in a 2 mL Eppendorf tube containing 700 µL of sterile saline by a sterile pestle followed by vortexing for 60 sec and plating of the supernatants in 3 different concentrations in sterile saline: undiluted, 10^−1^, 10^−2^.

For obtaining gut epithelium and digesta from the fish GI tract, the ventral fish surface was disinfected with 70% ethanol, after which the abdomen was carefully opened with sterile surgical scissors. For sampling digesta, the gut content was squeezed into a sterile 2 mL Eppendorf tube and 700 µL of sterile saline were added representing the undiluted (1:1) sample. Two serial dilutions (10^−1^, 10^−2^) were performed. Sampling of epithelium associated microbiota was conducted by thoroughly rinsing the emptied gut 3 times by sterile saline, then the gut was transferred into sterile Eppendorf tubes and serially diluted resulting in 3 different concentrations (10^0^, 10^−1^, 10^−2^). All three prepared dilutions from the fish gills, gut and digesta were plated on three different solid media (see Section 4.3). Water samples were directly plated (100 µL).

### 4.3. Isolation and Identification of Microorganisms

In order to isolate the broadest spectrum of associated microbiota, three different general media and a Miniaturized Culture Chip (MCC) device (Hoekmine BV, Utrecht, The Netherlands) were used. All three solid media, namely Tryptic Soy Broth (TSB12+5; Bacto Tryptic Soy Broth: 12 g·L^−1^, sodium chloride: 5 g·L^−1^), modified Wickerham medium (WSP15; Glucose monohydrate: 10 g·L^−1^, papain-digested peptone from soymeal: 5 g·L^−1^, malt extract: 3 g·L^−1^, yeast extract: 3 g·L^−1^, sodium chloride: 15 g·L^−1^) and Potato Dextrose Agar (PDA; potato infusion: 4 g·L^−1^, glucose monohydrate: 20 g·L^−1^, pH 5.6) media were prepared with 1.5% bacteriology grade agar (AppliChem GmbH, Darmstadt, Germany). Additionally, fish medium agar (FM) was developed from the fish gut contents and Baltic seawater to closely mimic the natural microenvironment of fish gut for application of the MCC. Petri dishes were inoculated with 100 µL aliquots from the prepared 10-fold dilutions and incubated at 22 °C in the dark. For isolation with the MCC, the FM plates were inoculated with 100 µL of the serial dilutions and the MCC was placed on top.

Cultures were checked weekly for 1.5 months and phenotypically distinct microbial colonies were transferred to fresh agar plates until pure cultures were obtained. Pure microbial strains were cryo-conserved at −80 °C using the Microbank System (Pro-Lab Diagnostics, Richmond Hill, Canada).

DNA was obtained either by following a freeze-and-thaw procedure for bacteria (transfer of cell material from well-grown culture to 100 µL DNA-free water, freezing overnight at −20 °C, followed by heat-shock for 15 min at 99 °C) or by mechanical cell lysis for fungi (transfer of cell material from well grown fungal mycelium to innuSPEED Lysis Tubes S (Analytik Jena, Jena, Germany) containing 400 µL DNA-free water, followed by shaking for 6 min at a frequency of 30 s^−1^ in a mixer mill MM 200 (Retsch, Hahn, Germany) and centrifugation of 8917 rpm for 5 min at room temperature). If those methods failed, isolation was repeated using the DNeasy Plant Mini Kit (Qiagen, Hilden, Germany) following the manufacturer’s guidelines. For molecular identification, the 16S rRNA gene (bacteria) or the ITS1-5.8S rRNA gene-ITS2 fragment (fungi) were PCR-amplified following as described before [92]. Sanger sequencing [23] of successfully amplified PCR products was conducted at LGC Genomics (Berlin, Germany) using primers 1387R for sequencing of bacterial and ITS1 for fungal DNA. Resulting partial sequences were trimmed by ChromasPro V1.33 (Technelysium Pty Ltd., South Brisbane, Australia) and compared to the National Center for Biotechnology Information (NCBI) nucleotide database using the BLAST (Basic local alignment search tool, [93]) algorithm. All sequences were submitted to NCBI Genbank and were assigned Accession Numbers ON782585-ON782643 (16S rRNA gene sequences and ON791469-ON791475 (ITS sequences) shown in Supplementary Table S1.

### 4.4. Cultivation

All selected isolates were grown in two media in solid (supplemented with 10 g·L^−1^ Difco Agar Noble (Becton Dickinson, Franklin Lakes, USA) and liquid culture regimes; bacteria were grown in Marine Broth/Agar (Ready-to-use mixture of Difco Marine Broth (Becton Dickinson, Franklin Lakes, USA): 37.4 g·L^−1^) and fungi in PDA (potato infusion: 4 g·L^−1^, glucose monohydrate: 20 g·L^−1^, pH 5.6). Selected isolates were regrown from cryo-conservation on solid medium. Subsequently, precultures were prepared for liquid (50 mL) and solid regime cultivation and incubated for 7 days (bacteria) and 14 days (fungi) at 22 °C in the dark on Marine Agar (bacteria) and PDA (fungi) Liquid precultures were additionally shaken (VKS-75 control, Edmund Bühler, Hechingen, Germany) at 120 rpm to provide aeration.

Liquid main cultures were inoculated using cell material from the respective liquid pre-culture with an optical density (OD_600_) of 0.01 in 500 mL medium (2 L Erlenmayer flasks). Accordingly, solid main cultures were inoculated from the solid pre-culture plates and grown in duplicates of 10 plates each. All cultures were incubated in the dark at 22 °C, again, liquid cultures were shaken at 120 rpm. Bacteria were grown for 7 and fungi for 14 days.

### 4.5. Extraction

For extraction of solid media cultures, agar plates were cut into pieces and transferred into 1 L glass bottles using a flat spatula. Subsequently, 400 mL of EtOAc (Pestinorm, VWR Chemicals, Leuven, Belgium) were added. The mixtures were homogenized using an Ultra Turrax (IKA-Werke, Staufen, Germany) at 13,000 rpm for 30 s and stored overnight on a rotary shaker at 120 rpm and 22 °C. On the next day, the liquid phase was poured into a separatory funnel and a liquid/liquid partitioning was created by adding an equal amount of Mili-Q water (Arium Lab water systems, Sartorius, Goettingen, Germany). Extensive shaking by hand ensured mixing of the solvents. When phase separation was visible, the water phase was disposed, to remove highly polar metabolites or medium constituents such as sugars and salts. The EtOAc partition was kept in a round bottom flask. Meanwhile, another 400 mL of EtOAc were added to the remaining agar for a second extraction. The mixture was sonicated for 15 min to facilitate metabolite dissolution (Sonorex Super RK 106, Bandelin Electronic GmbH & Co. KG, Berlin, Germany), subsequently the previous steps were repeated. Finally, both organic phases were then combined and dried on a rotary evaporator (Laborota 4000 efficient, Heidolph Instruments GmbH & CO. KG, Schwabach, Germany) at 150 rpm and 40 °C water bath temperature and a vacuum of 140 mbar. The crude extract was dissolved in 3 mL of UPLC/MS grade MeOH and filtered through a 0.2 μm PTFE filter (VWR International, Darmstadt, Germany) into pre-weighed amber glass vials. Finally, all samples were dried under a nitrogen flow at 40 °C, weighed again and stored at -20 °C. Likewise media blanks were prepared for comparison.

Liquid cultures were mixed with the same volume (500 mL) of EtOAc in a separatory funnel and shaken rigorously by hand for 1 min. Fungal strains were additionally treated with an ultra-turrax at 13,000 rpm to break the cells. Subsequently, the lower (water) phase was collected for a second extraction, while the organic phase was cleaned twice with MiliQ water and kept in a round bottom flask. Both organic layers were eventually combined and evaporated on a rotary evaporator. Finally, all crude extracts were dissolved in MeOH, filtered into amber glass vials, dried and stored at −20 °C as described above for the solid extraction. Media blanks were treated equally.

### 4.6. UPLC-QToF-MS/MS-Based Metabolome Analyses

The EtOAc extracts and controls (media blanks and solvents) were analysed on an ACQUITY UPLC I-Class System coupled to the Xevo G2-XS QToF Mass Spectrometer (Waters^®^, Milford, MA, USA) equipped with an electrospray ionization (ESI) source operating with a mass range of *m/z* 50–1600 Da. Each extract was dissolved in MeOH and filtered through a 0.2 µm PTFE syringe filter to give a system equipped with an Acquity UPLC HSS T3 column (High Strength Silica C18, final concentration of 0.1 mg/mL, 1.8 µm, 100 × 2.1 mm I.D., Waters^®^) operating at 40 °C. Injection volume of samples was 0.6 µL. The chemical analysis was processed with a binary LC solvent system controlled by MassLynx^®^ (version 4.1) using mobile phase A (99.9%) water/0.1% formic acid (ULC/MS grade) and B 99.9% ACN/0.1% formic acid (ULC/MS grade), pumped at a rate of 0.4 mL/ min with the following gradient: initial, 1% B; 0.0–11.5 min to 100% B; 11.5–12.5 min 100% B, and a column reconditioning phase to 1% B until 16 min. ESI conditions were set as follows: capillary voltage at 3 kV, sample cone voltage at 50.0 V, source temperature at 150 °C, desolvation temperature at 550 °C, cone gas flow in 50 L/h and desolvation gas flow in 1200 L/h. MS/MS setting was a ramp collision energy (CE): low CE from 6 eV to 60 eV and high CE from 9–80 eV. Media extracts and solvent (methanol) were injected as controls. MassLynx^®^ (Waters^®^, V4.1) was used to analyze the recorded MS^1^ and MS^2^ data. Each run was repeated three times.

The raw data were converted to mzXML file format using MSconvert (version 3.0.10051, Vanderbilt University, Nashville, TE, USA). The resulting mzXML data were processed in MZmine2 (version 2.32, [94,95]). Mass detection was set to a noise level of 100 and 10 counts for MS^1^ and MS^2^, respectively. The chromatogram was built using peak lists with a scan retention time from 0.00 min to 13.0 min, and a minimum peak height of 30,000 counts. The chromatogram was deconvoluted with the baseline algorithm. For MS^2^ scan pairing, the *m/z* range was set to 0.5 Da as well as retention time range to 0.1 min. Minimal cosine similarity score was set to 0.7 and a minimum of 6 fragment ion peaks needed to match. Deisotoping of the chromatogram was achieved by the isotope peak grouper algorithm with *m/z* tolerance of 0.01 ppm and RT tolerance 0.3 min. The aligned peak list was filtered to exclude peaks derived from solvent and peaks with *m/z* lower than 141 Da. The peak IDs were reset last and the peak list was exported as .mgf file for GNPS analysis. The reset peak list was exported as .CSV table file to assist the creation of molecular networks.

A molecular network was created with the feature-based molecular networking (FBMN) workflow on GNPS [24]. The data was filtered by removing all MS/MS fragment ions within ±17 Da of the precursor *m/z*. MS/MS spectra were window filtered by choosing only the top 6 fragment ions in the ±50 Da window throughout the spectrum. The precursor ion mass tolerance was set to 0.02 Da and the MS/MS fragment ion tolerance to 0.02 Da. A molecular network was then created where edges were filtered to have a cosine score above 0.7 and more than 6 matched peaks. Further, edges between two nodes were kept in the network if and only if each of the nodes appeared in each other’s respective top 10 most similar nodes. Finally, the maximum size of a molecular family was set to 100, and the lowest scoring edges were removed from molecular families until the molecular family size was below this threshold. The spectra in the network were then searched against GNPS spectral libraries [96]. The library spectra were filtered in the same manner as the input data. All matches kept between network spectra and library spectra were required to have a score above 0.7 and at least 6 matched peaks. The molecular networks were visualized using Cytoscape software [97]. Molecular formula predictions were done with MassLynx version 4.1. for annotation of parent ions. Predicted molecular formulae were searched against databases such as Dictionary of Natural Product (http://dnp.chemnetbase.com, accessed on 10 January 2022), NP Atlas (https://www.npatlas.org, accessed on 10 January 2022) and SciFinder (https://scifinder.cas.org, accessed on 11 January 2022). Dereplicated peak ions were further checked manually or by comparing the experimental fragments to in-silico fragments generated from the CFM-ID web server [98]. The molecular networking job on GNPS can be found at https://gnps.ucsd.edu/ProteoSAFe/status.jsp?task=36c108371b1f41439b102a8ab75f1fce (accessed on 30 January 2022). 

### 4.7. Bioactivity Assessments

Antimicrobial assays were performed in 96-well plates using (i) the ESKAPE panel of human nosocomial pathogens, including the Gram-positive bacteria *Enterococcus faecium* DSM 20477 and the methicillin-resistant *Staphylococcus aureus* DSM 18827, and the gram-negative bacteria *Klebsiella pneumoniae* DSM 30104, *Acinetobacter baumannii* DSM 30007, *Pseudomonas aeruginosa* DSM 1128 and *Escherichia coli* DSM 1576, (ii) the fish pathogenic bacteria *Lactococcus garviae* DSM 20684, *Vibrio anguillarum* DSM 21597 and *Vibrio ichthyoenteri* DSM 14397 (iii) human pathogenic yeasts *Candida albicans* DSM 1386 and *Cryptococcus neoformans* DSM 6973. All test strains were purchased from Leibniz Institute DSMZ (Braunschweig, Germany). The screening was performed as described previously [99]. Briefly, the extracts were dissolved in DSMO and transferred in duplicates into a microplate for a final assay concentration of 100 µg/mL. Five ESKAPE panel pathogens were cultivated in TSB medium (1.2% tryptic soy broth, 0.5% NaCl). *Enterococcus faecium* and *L. garviae* were cultured in M92 medium (3% trypticase soy broth, 0.3% yeast extract, pH 7.0–7.2). *Vibrio* spp. were grown in TM medium (3% sea salt mixture Instant Ocean, 0.5% peptone, 0.1% yeast extract) while the yeasts were cultivated in M186 (1% glucose, 0.5% peptone from soybean meal, 0.3% yeast extract, 0.3% malt extract). From each test organism an overnight culture was prepared and diluted to an optical density (600 nm) of 0.01–0.03. A 200 µL of the inoculum was added to each well of the microplate. The ESKAPE panel, *C. albicans* and *L. garviae* were incubated for 5 h at 37 °C and shaking at 200 rpm, while *E. faecium* and *L. garviae* were not shaken. All other test organisms were grown for 6 h at 28 °C and shaking at 200 rpm. After incubation, 10 µL of a resazurin solution (0.3 mg/mL phosphate-buffered saline) was added to the microplate. After another incubation for 5–60 min at room temperature the fluorescence signal (560 nm / 590 nm) was measured using the microplate reader (Tecan Infinite M200, Tecan, Männedorf, Switzerland). For *E. faecium* and *L. garviae* the pH indicator bromocresol purple was used to determine the acidification caused by growing and the absorbance was measured (600 nm/690 nm reference). For *C. neoformans* the absorbance at 600 nm was detected. The resulting values were compared with a positive control, chloramphenicol (*S. aureus, E. coli, K. pneumoniae, Vibrio* spp.), ampicillin (*E. faecium, L. garviae),* polymyxin B (*P. aeruginosa*) doxycycline (*A. baumannii*), nystatin (*C. albicans*) and amphotericin B (*C. neoformans*), plus a negative control (no compound) on the same plate. For IC_50_ determinations, a dilution series of the extracts were transferred into the microplate. The IC_50_ values were calculated by Excel as the concentration that show 50% inhibition of viability on the basis of a negative control.

## Figures and Tables

**Table 1:** The IC_50_ values (µg/mL) of bioactive extracts in different media and culture regimes. The table does not include the inactive strains. Isolate codes: PG: Plaice Gill, PI: Plaice Intestine Epithelium, PS: Plaice Stomach Epithelium, PID: Plaice Intestine Digesta, PSD: Plaice Stomach Digesta, W: Water; MCC: Microchip isolation, -B: Bacterium and -F: Fungus. n.g. No or very poor growth. Vi: *Vibrio ichthyoenteri* Lg: *Lactococcus garvieae*, MRSA: Methillicin-resistant *Staphylococcus aureus*, Ef: *Enterococcus faecium*. Positive controls: Chloramphenicol (Vi and MRSA), Ampicillin (Lg and Ef).

Code	Taxonomical ID	Origin	Medium/ Regime	Vi	Lg	MRSA	Ef
PG1-B	*Shewanella baltica*	Gill	MA solid	>100	>100	20.2	26.4
			MB liquid	>100	41.7	18.8	8.8
PG10-B	*Chryseobacterium carnis*	Gill	MA solid	18.6	>100	39.0	41.9
			MB liquid	>100	>100	>100	51.0
PG11-B	*Microbacterium* sp.	Gill	MA solid	>100	>100	45.9	86.6
			MB liquid	5.7	>100	>100	>100
PG12-B	*Vibrio aestuarianus*	Gill	MA solid	17.2	27.8	19.1	34.4
			MB liquid	43.9	>100	61.0	43.4
PID21-B	*Bacillus* sp.	Gut (Intestine) Digesta	MA solid	4.7	>100	10.2	>100
			MB liquid	28.4	>100	5.9	>100
PI1-B	*Bacillus* sp.	Gut (Intestine) Epithelium	MA solid	>100	14.7	18.5	15.0
			MB liquid	>100	54.2	22.1	11.6
PI2-B	*Vibrio* sp.	Gut (Intestine) Epithelium	MA solid	>100	14.1	15.8	17.5
			MB liquid	>100	>100	36.4	9.0
PI8-B	*Pseudoalteromonas* sp.	Gut (Intestine) Epithelium	MA solid	>100	28.3	21.2	50.4
			MB liquid	>100	36.6	8.1	8.2
PS3-B	*Psychrobacter* sp.	Gut (Stomach) Epithelium	MA solid	>100	>100	23.3	28.9
			MB liquid	>100	>100	11.4	7.5
MCC-PID1-B	*Photobacterium* sp.	Gut (Intestine) Digesta	MA solid	>100	40.2	35.0	69.0
			MB liquid	n.g.	n.g.	n.g.	n.g.
PSD4-B	*Shewanella* *colwelliana*	Gut (Stomach) Digesta	MA solid	>100	25.4	29.1	30.8
			MB liquid	n.g.	n.g.	n.g.	n.g.
PID2-B	*Shewanella aestuarii*	Gut (Intestine) Digesta	MA solid	n.g.	n.g.	n.g.	n.g.
			MB liquid	>100	>100	6.8	4.9
PI9-F	*Aureobasidium* *pullulans*	Gut (Intestine) Epithelium	PDA solid	>100	22.6	40.8	30.7
			PDA liquid	>100	>100	8.9	3.3
	Positive control			0.4	0.5	1.5	0.2

## Data Availability

The data may be available by the corresponding author.

## Supplementary Materials

The following supporting information can be downloaded at: https://www.mdpi.com/article/10.3390/md20090573/s1. Supplementary Figure S1: Diversity and distribution of bacterial isolates from plaice gill, gut and sea water reference at family level. SW: Sea water, PG: Plaice Gill, PS: Plaice Stomach Epithelium, PSD: Plaice Stomach Digesta, PI: Plaice Intestine Epithelium, PID: Plaice Intestine Digesta. Figure S2: Diversity and distribution of plaice-derived bacteria recovered from different media at family level. TSB: Tryptic Soy medium, WSP: Wickerham medium, PDA: Potato Dextrose medium, FM: Fish medium. Figure S3: Diversity and distribution of 11 seawater-derived bacterial isolates at A. family and B. genus level recovered from different media. TSB: Tryptic Soy medium, WSP: Wickerham medium, PDA: Potato Dextrose medium, FM: Fish medium. Figure S4: Taxonomic distribution of 13 bioactive microorganisms at phylum level. Note that the Figure contains also one bioactive fungal isolate (A. pullulans PI9-F belonging to Ascomycota, coloured in red). Table S1: BLAST results from comparison of 66 microbial strains isolated from European plaice and seawater reference to the NCBI nucleotide database incl. Genbank accession numbers assigned to the isolates; Table S2: Identification of 66 microbial strains isolated from European plaice and seawater reference to the highest possible taxonomic level; Table S3: Biological activity of all 23 selected plaice-associated microorganisms. Tables S4–S15: Putative annotation of metabolites detected in all bioactive extracts.
