# Supplementary material for: Gut and Gill-Associated Microbiota of the Flatfish European Plaice (Pleuronectes platessa): Diversity, Metabolome and Bioactivity against Human and Aquaculture Pathogens
Source: Mar Drugs. 2022 Sep 9;20(9):573. doi: 10.3390/md20090573 (PMC9500656; doi:10.3390/md20090573)
Supplement: Supplementary file 1 [file marinedrugs-20-00573-s001.zip › marinedrugs-1848971-SI.pdf]

Supplementary Information

Gut and Gill-Associated Microbiota of the Flatfish European plaice (*Pleuronectes platessa)*: Diversity, Metabolome and Bioactivity against Human and Aquaculture Pathogens

Marjan Ghotbi [1,†], Ole Kelting [1,†], Martina Blümel [1] and Deniz Tasdemir [1,2,*]

1 GEOMAR Centre for Marine Biotechnology (GEOMAR-Biotech), Research Unit Marine Natural Product Chemistry, GEOMAR Helmholtz Centre for Ocean Research Kiel, Am Kiel-Kanal 44, 24106 Kiel, Germany

2 Faculty of Mathematics and Natural Sciences, Kiel University, Christian-Albrechts-Platz 4, 24118 Kiel, Germany

* Correspondence: dtasdemir@geomar.de; Tel.: +49-431-600-4430

† These authors contributed equally to this work.

Supplementary Figures

Figure S1. Diversity and distribution of bacterial isolates from plaice gill, gut and sea water reference at family level

Figure S2. Diversity and distribution of plaice-derived bacteria recovered from different media at family level

Figure S3. Diversity and distribution of 11 seawater-derived bacterial isolates recovered from different media on A) family and B) genus level

Figure S4. Taxonomic distribution of 13 bioactive microorganisms at phylum level

Supplementary Tables

Table S1. BLAST results from comparison of 66 microbial strains isolated from European plaice and seawater reference to the NCBI nucleotide database incl. Genbank accession numbers assigned to the isolates

Table S2. Identification of 66 microbial strains isolated from European plaice and seawater reference to the highest possible taxonomic level

Table S3. Biological activity of all 23 selected plaice-associated microorganisms

Table S4. Putative annotation of metabolites detected in *Bacillus* sp. PID21-B

Table S5. Putative annotation of metabolites detected in *Bacillus* sp. PI1-B

Table S6. Putative annotation of metabolites detected in *Microbacterium* sp. PG11-B

Table S7. Putative annotation of metabolites detected in *Chryseobacterium carnis* PG10-B

Table S8. Putative annotation of metabolites detected in *Shewanella baltica* PG1-B

Table S9. Putative annotation of metabolites detected in *Shewanella aestuarii* PID2-B

Table S10. Putative annotation of metabolites detected in *Shewanella colwelliana* PSD4-B

Table S11. Putative annotation of metabolites detected in *Psychrobacter* sp. PS3-B

Table S12. Putative annotation of metabolites detected in *Vibrio aestuarianus* PG12-B

Table S13. Putative annotation of metabolites detected in *Vibrio* sp. PI2-B

Table S14. Putative annotation of metabolites detected in *Pseudoalteromonas* sp. PI8-B

Table S15. Putative annotation of metabolites detected in *Aureobasidium pullulans* PI9-F

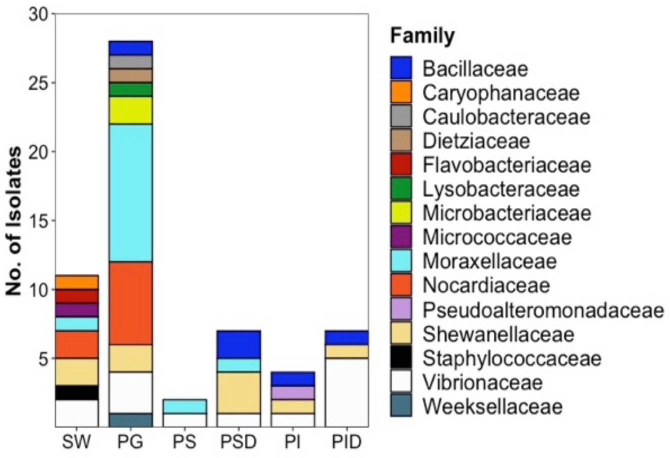

Figure S1. Diversity and distribution of bacterial isolates from plaice gill, gut and sea water reference at family level. SW: Sea water, PG: Plaice Gill, PS: Plaice Stomach Epithelium, PSD: Plaice Stomach Digesta, PI: Plaice Intestine Epithelium, PID: Plaice Intestine Digesta.

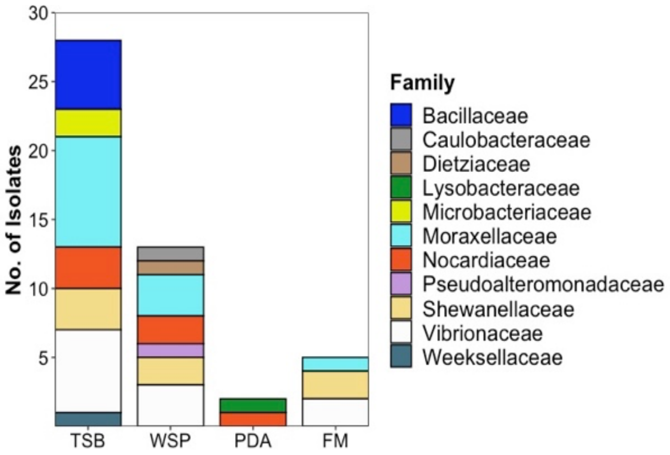

Figure S2. Diversity and distribution of plaice-derived bacteria recovered from different media at family level. TSB: Tryptic Soy medium, WSP: Wickerham medium, PDA: Potato Dextrose medium, FM: Fish medium.

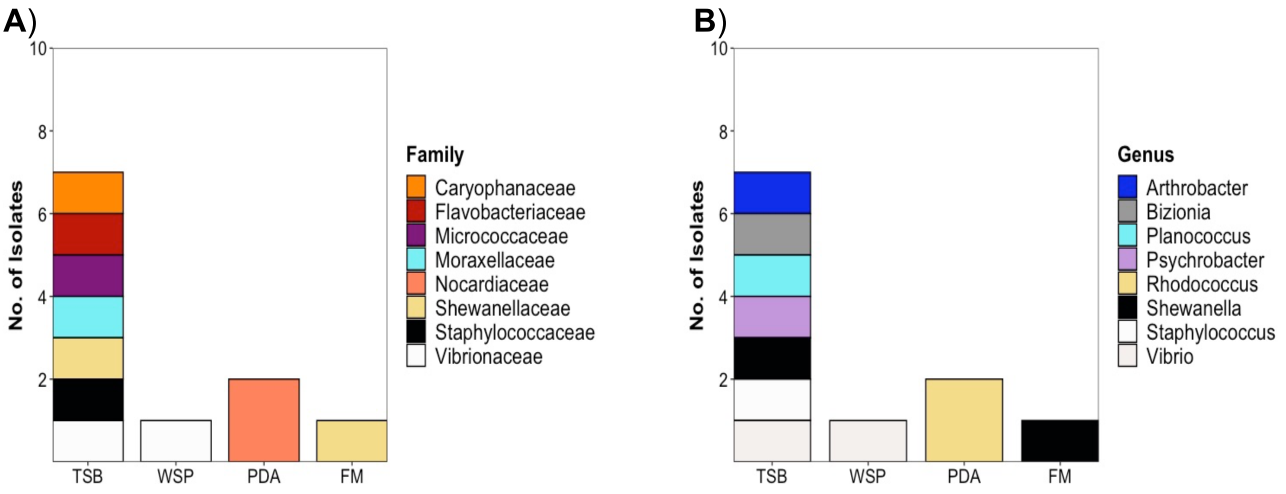

Figure S3. Diversity and distribution of 11 seawater-derived bacterial isolates at **A**. family and **B**. genus level recovered from different media. TSB: Tryptic Soy medium, WSP: Wickerham medium, PDA: Potato Dextrose medium, FM: Fish medium

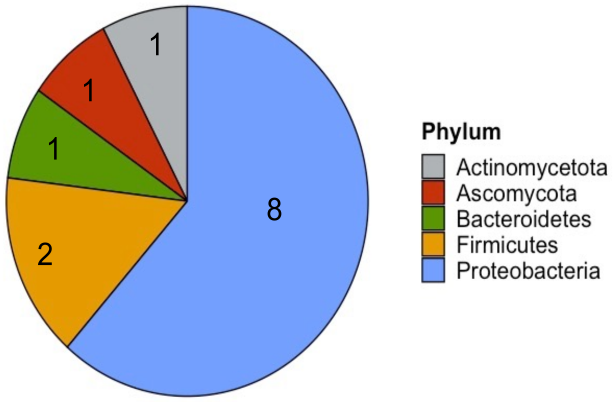

Figure S4. Taxonomic distribution of 13 bioactive microorganisms at phylum level. Note that the Figure contains also one bioactive fungal isolate (*A. pullulans* PI9-F belonging to Ascomycota, coloured in red).

Table S1. BLAST results from comparison of 66 microbial strains isolated from European plaice and the seawater reference to the NCBI nucleotide database. Given are the first 3 hits from two BLAST searches, one against all entries and the second against type strains only. Isolate codes: **PG**: Plaice Gut, **PS**: Plaice Stomach, **PSD**: Plaice Stomach Digesta, **PI**: Plaice Intestines, **PID**: Plaice Intestine Digesta, **W**: Water reference, **MCC**: Isolation on Microchip using Fish Medium, -B: Bacterium, -F: Fungus

Strain ID	Source	Seq length (bp)	three closest relatives according to nucleotide BLAST (accession date June 13, 2022)	NCBI Accession No. of closest relatives	simi-larity (%)	query cover (%)	three closest relatives according to nucleotide BLAST, only TYPE strains (accession date June 13, 2022)	NCBI Accession No of closest relatives	simi-larity (%)	query cover (%)	NCBI Genbank Acc. No
PG1-B	Gill	914	Shewanella baltica strain CD-1<br>Shewanella baltica strain SB06<br>Shewanella baltica strain SN_11	MN220604.1<br>KT716385.1<br>KR088613.1	100<br>100<br>100	100<br>100<br>100	Shewanella arctica type strain 40-3T<br>Shewanella hafniensis strain P010<br>Shewanella hafniensis strain NBRC 100975	AJ877256.1<br>NR_041296.1<br>NR_113967.1	99.9<br>98.5<br>97.6	100<br>100<br>100	ON782585
PG2-B	Gill	994	Vibrio anguillarum strain VIB16<br>Vibrio anguillarum strain 2NS-PRS3-a2<br>Vibrio anguillarum strain VIB12	KF150783.1<br>MG264177.1<br>CP023310.1	99.9<br>99.7<br>99.7	100<br>100<br>100	Shewanella arctica type strain 40-3T<br>Shewanella hafniensis strain P010<br>Shewanella hafniensis strain NBRC 100975	CP022741.1<br>CP010084.1<br>NR_148247.1	98.8<br>98.8<br>98.8	100<br>100<br>100	ON782586
PG4-B	Gill	871	Photobacterium kishitanii Taigaleon<br>Photobacterium sp. strain HS256<br>Photobacterium sp. strain HS075	LC421662.1<br>MH269407.1<br>MH269398.1	100<br>100<br>100	100<br>100<br>100	Photobacterium carnosum strain TMW 2.2021<br>Photobacterium piscicola strain NCCB 100098<br>Photobacterium phosphoreum strain NBRC 103031	NR_156814.1<br>NR_125679.1<br>NR_114184.1	100<br>99.9<br>100	100<br>100<br>99	ON782587
PG5-B	Gill	881	Psychrobacter faecalis strain NC7<br>Psychrobacter faecalis strain 96D12<br>Psychrobacter pulmonis strain 96D7	MT269580.1<br>MT013488.1<br>MT013485.1	100<br>100<br>100	100<br>100<br>100	Psychrobacter pulmonis strain CCUG 46240<br>Psychrobacter faecalis strain DSM 14664<br>Psychrobacter pulmonis strain S-606	NR_118026.1<br>NR_118025.1<br>NR_028974.1	100<br>99.8<br>99.8	100<br>100<br>100	ON782588
PG6-B	Gill	786	Psychrobacter glacincola strain 38-1<br>Psychrobacter sp. strain P151-L01a<br>Psychrobacter sp. strain AsK2	MN326773.1<br>MN043902.1<br>MK560045.1	99.87<br>99.87<br>99.87	100<br>100<br>100	Psychrobacter immobilis strain NBRC 15733<br>Psychrobacter immobilis strain ATCC 43116<br>Psychrobacter cibarius strain JG-219	NR_113805.1<br>NR_118027.1<br>NR_043057.1	99.87<br>99.87<br>99.87	100<br>100<br>100	ON782589
PG8-B	Gill	703	Psychrobacter faecalis strain NC7<br>Psychrobacter pulmonis strain 190306H131<br>Psychrobacter faecalis strain 96D12	MT269580.1<br>MT225754.1<br>MT013488.1	100<br>100<br>100	100<br>100<br>100	Psychrobacter pulmonis strain CCUG 46240<br>Psychrobacter faecalis strain DSM 14664<br>Psychrobacter pulmonis strain S-606	NR_118026.1<br>NR_118025.1<br>NR_028974.1	100<br>99.9<br>99.9	100<br>100<br>100	ON782590
PG9-B	Gill	827	Psychrobacter maritimus strain JM52<br>Psychrobacter sp. strain RSAP27<br>Psychrobacter sp. strain RSAP9	MN758812.1<br>MH348992.1<br>MH348978.1	100<br>100<br>100	100<br>100<br>100	Psychrobacter maritimus strain Pi2-20<br>Psychrobacter namhaensis strain SW-242<br>Psychrobacter namhaensis strain SW-242	NR_027225.1<br>MW227500.1<br>NR_043141.1	100<br>99.5<br>99.5	100<br>100<br>100	ON782591
PG10-B	Gill	875	Kaistella carnis strain G0081<br>Chryseobacterium sp. CH1(2015)<br>Chryseobacterium sp. B-G-R2A3	CP034159.1<br>KP188538.1<br>HM629415.1	100<br>100<br>100	100<br>100<br>100	Kaistella carnis strain G0081<br>Kaistella carnis strain G81<br>Kaistella yonginensis strain HMD1043	CP034159.1<br>NR_126255.1<br>NR_108572.1	100<br>99.8<br>98.2	100<br>100<br>100	ON782592

PG11-B	Gill	601	Microbacterium sp. strain YHDJ3	ON351061.1	100	100	Microbacterium algeriense strain G1	MK480726.1	100	100	ON782593
			Microbacterium hydrocarbonoxydans strain J14-3-1	ON337519.1	100	100	Microbacterium phyllosphaerae strain DSM 13468 (T)	MK424292.1	100	100	
			Microbacterium sp. SSW1-36	CP078077.1	100	100	Microbacterium hydrocarbonoxydans strain NBRC 103074 (T)	MK424288.1	100	100	
PG12-B	Gill	920	Vibrio aestuarianus strain 15_075_1T2	MK307696.1	100	100	Vibrio aestuarianus subsp. cardii strain 12/122 3T3	MK307684.1	100	100	ON782594
			Vibrio aestuarianus strain 15_064_4T2	MK307695.1	100	100	Vibrio aestuarianus strain NBRC 15629	NR_113780.1	100	100	
			Vibrio aestuarianus strain 15_061_5T2	MK307693.1	100	100	Vibrio aestuarianus strain CAIM 592	MT759936.1	100	100	
PG14-B	Gill	892	Psychrobacter maritimus strain JM52	MN758812.1	99.9	100	Psychrobacter maritimus strain Pi2-20	NR_027225.1	99.7	100	ON782595
			Psychrobacter maritimus strain 24d-S2	MN062085.1	99.9	100	Psychrobacter namhaensis strain SW-242	MW227500.1	99.5	100	
			Psychrobacter maritimus strain 16d-S4	MN062076.1	99.9	100	Psychrobacter pulmonis strain CCUG 46240	NR_118026.1	99.1	100	
PG15-B	Gill	737	Stenotrophomonas rhizophila strain LA-3	MT631997.1	100	100	Stenotrophomonas nematodicola culture CPCC:101271	MT126327.1	100	100	ON782596
			Stenotrophomonas sp. strain B10	MT576573.1	100	100	Stenotrophomonas rhizophila strain e-p10	NR_121739.1	100	100	
			Stenotrophomonas sp. strain WA7-2-5	MH341940.1	100	100	Stenotrophomonas rhizophila strain DSM14405	CP007597.1	100	100	
PG16-B	Gill	759	Rhodococcus sp. strain YF-9	MT631993.1	100	100	Rhodococcus erythropolis strain: JCM 3201	AB429553.1	100	100	ON782597
			Rhodococcus qingshengii strain cqsV23	MN826591.1	100	100	Rhodococcus erythropolis strain DSM 43066	KJ476725.1	100	100	
			Rhodococcus qingshengii strain cqsV7	MN826576.1	100	100	Nocardia coeliaca strain DSM 44595	NR_104776.1	100	100	
PG17-B	Gill	808	Psychrobacter maritimus strain JM52	MN758812.1	100	100	Psychrobacter maritimus strain Pi2-20	NR_027225.1	99.8	100	ON782598
			Psychrobacter maritimus strain 24d-S2	MN062085.1	100	100	Psychrobacter namhaensis strain SW-242	MW227500.1	99.5	100	
			Psychrobacter maritimus strain 16d-S4	MN062076.1	100	100	Psychrobacter namhaensis strain SW-242	NR_043141.1	99.1	100	
PG18-B	Gill	766	Rhodococcus sp. strain 2792	MT586011.1	100	100	Rhodococcus cerastii strain C5	NR_117103.1	99.1	100	ON782599
			Rhodococcus yunnanensis strain PAMC 27323	MT555344.1	100	100	Rhodococcus cercidiphylli strain YIM 65003	NR_116275.1	99.1	100	
						100					

			Rhodococcus yunnanensis strain PAMC 27319	MT555341.1	100		Rhodococcus yunnanensis strain YIM 70056	NR_043009.1	99.1	100	
PG19-B	Gill	980	Rhodococcus sp. FI 1025	JQ691550.1	100	100	Rhodococcus fascians strain CF17	NR_037021.1	99.2	100	ON782600
			Rhodococcus yunnanensis strain OsEnb_PLM_L24	MN889263.1	99.6	100	Rhodococcus fascians strain ATCC 12974	NR_119126.1	99.0	100	
			Rhodococcus sp. strain O1	MT012171.1	99.6	100	Rhodococcus sovatensis strain H004	NR_156055.1	99.0	100	
PG20-B	Gill	772	Bacillus licheniformis strain T-5-9-2	ON331910.1	100	100	Bacillus subtilis subsp. inaquosorum strain BGSC 3A28	MK183752.1	100	100	ON782601
			Bacillus licheniformis strain HBUAS664885	ON306832.1	100	100	Bacillus licheniformis strain DSM 13	MN396732.1	100	100	
			Bacillus licheniformis strain HBUAS664883	ON306830.1	100	100	Bacillus licheniformis strain ATCC 14580	CP034569.1	100	100	
PG25-B	Gill	850	Rhodococcus sp. strain YF-9	MT631993.1	100	100	Rhodococcus erythropolis strain: JCM 3201	AB429553.1	100	100	ON782602
			Rhodococcus qingshengii strain cqsV23	MN826591.1	100	100	Rhodococcus erythropolis strain DSM 43066	KJ476725.1	100	100	
			Rhodococcus qingshengii strain cqsV7	MN826576.1	100	100	Nocardia coeliaca strain DSM 44595	NR_104776.1	100	100	
PG26-B	Gill	820	Rhodococcus sp. strain 23770	MT357195.1	100	100	Rhodococcus fascians strain CF17	NR_037021.1	100	100	ON782603
			Rhodococcus sp. strain PN2-B08P5-12	MK638437.1	100	100	Rhodococcus fascians strain ATCC 12974	NR_119126.1	99.8	100	
			Rhodococcus sp. strain PN2-B07P1-10	MK638436.1	100	100	Rhodococcus cerastii strain C5	NR_117103.1	99.6	100	
PG31-B	Gill	540	Rhodococcus sp. strain 2792	MT586011.1	100	100	Rhodococcus cercidiphylli strain: YIM 65003, clone: S1-903	LC130641.1	99.4	100	ON782604
			Rhodococcus yunnanensis strain PAMC 27323	MT555344.1	100	100	Rhodococcus cerastii strain C5	NR_117103.1	99.4	100	
			Rhodococcus yunnanensis strain PAMC 27319	MT555341.1	100	100	Rhodococcus cercidiphylli strain YIM 65003	NR_116275.1	99.4	100	
PG32-B	Gill	431	Psychrobacter submarinus strain ACBC051	MK123477.1	100	100	Psychrobacter maritimus strain Pi2-20	NR_027225.1	100	100	ON782605
			Psychrobacter submarinus strain QS172	MK439598.1	100	100	Psychrobacter submarinus strain KMM 225	NR_025457.1	100	100	
			Psychrobacter marincola strain QS123	MK439597.1	100	100	Psychrobacter marincola strain KMM 277	NR_025458.1	99.8	100	
PG37-B	Gill	645	Psychrobacter maritimus strain JM52	MN758812.1	100	100	Psychrobacter maritimus strain Pi2-20	NR_027225.1	100	100	ON782606

			Psychrobacter sp. strain RSAP27<br>Psychrobacter sp. strain RSAP9	MH348992.1<br>MH348978.1	100<br>100	100<br>100	Psychrobacter submarinus strain KMM 225<br>Psychrobacter marincola strain KMM 277	NR_025457.1<br>NR_025458.1	100<br>100	99<br>98	
PG38-B	Gill	440	Psychrobacter sp. strain PL 19<br>Psychrobacter cryohalolentis strain 4_KNS_9_Sed_A2<br>Psychrobacter cryohalolentis strain 4_KNS_9_Sed_Z3	MT594461.1<br>MT309525.1<br>MT309524.1	99.77<br>99.77<br>99.77	100<br>100<br>100	Psychrobacter cryohalolentis K5<br>Psychrobacter arcticus strain 273-4<br>Psychrobacter okhotskensis strain MD17	NR_075055.1<br>NR_075054.1<br>MW228834.1	99.77<br>99.77<br>99.77	100<br>100<br>100	ON782607
PG39-B	Gill	406	Bacterium strain 8WHB1<br>Brevundimonas sp. strain A1P056<br>Brevundimonas sp. strain E	MT318440.1<br>MN989045.1<br>MN722460.1	100<br>100<br>100	100<br>100<br>100	Brevundimonas mediterranea strain V4.BO.10<br>Brevundimonas bullata strain HAMBI 262<br>Brevundimonas bullata strain IAM 13153(T)	NR_037108.1<br>LT899984.1<br>ON150872.1	100<br>99.5<br>99.5	100<br>100<br>100	ON782608
PG42-B	Gill	841	Dietzia alimentaria 72<br>Actinobacterium CH13i<br>Uncultured bacterium clone TSJG-5	NR_117306.1<br>FJ164056.1<br>KT905823.1	100<br>99.9<br>99.9	100<br>100<br>100	Dietzia alimentaria 72<br>Dietzia maris strain DSM 43672<br>Dietzia maris strain DSM 43672	NR_117306.1<br>MW578396.1<br>NR_118596.1	100<br>99.5<br>99.5	100<br>100<br>100	ON782609
PG43-B	Gill	645	Microbacterium algeriense strain 10<br>Microbacterium oxydans strain PD3N6a<br>Microbacterium phyllosphaerae strain PD3N6	ON428502.1<br>ON406315.1<br>ON406314.1	100<br>100<br>100	100<br>100<br>100	Microbacterium algeriense strain G1<br>Microbacterium phyllosphaerae strain DSM 13468 (T)<br>Microbacterium hydrocarbonoxydans strain NBRC 103074 (T)	MK480726.1<br>MK424292.1<br>MK424288.1	100<br>100<br>100	100<br>100<br>100	ON782610
MCC-PG1-B	Gill	873	Shewanella aestuarii strain Hal007<br>Shewanella aestuarii strain Hal006<br>Shewanella sp. AB411d	MT406388.1<br>MT406387.1<br>FR821223.1	100<br>100<br>100	100<br>100<br>100	Shewanella aestuarii strain JCM 17801<br>Shewanella aestuarii strain SC18<br>Shewanella glacialimarina strain TZS-4	MT760375.1<br>NR_135728.1<br>CP041216.1	99.20<br>99.08<br>98.28	100<br>100<br>100	ON782611
MCC-PG2-B	Gill	870	Psychrobacter sp. strain RSAP9<br>Psychrobacter sp. strain RSAP27<br>Psychrobacter maritimus strain HM-2	MH348978.1<br>MH348992.1<br>MH550141.1	100<br>100<br>100	100<br>99<br>99	Psychrobacter maritimus strain Pi2-20<br>Psychrobacter namhaensis strain SW-242<br>Psychrobacter okhotskensis strain MD17	NR_027225.1<br>MW227500.1<br>MW228834.1	100<br>99.2<br>99.1	99<br>100<br>100	ON782612
PS3-B	Stomach Epithelium	450	Unidentified marine bacterioplankton clone P3-4B_45<br>Psychrobacter sp. P11-S-4<br>Psychrobacter sp. strain PL 19	KC001357.1<br>EU016159.1<br>MT594461.1	95.33<br>95.33<br>95.11	100<br>100<br>100	Psychrobacter cryohalolentis K5<br>Psychrobacter arcticus strain 273-4<br>Psychrobacter okhotskensis strain MD17	NR_075055.1<br>NR_075054.1<br>MW228834.1	95.11<br>95.11<br>95.11	100<br>100<br>100	ON782613
MCC-PS2 -B	Stomach Epithelium	722	Photobacterium sp. strain TH13<br>Photobacterium sp. strain TH9<br>Photobacterium sp. strain TH8	MN049756.1<br>MN049752.1<br>MN049751.1	100<br>100<br>100	100<br>100<br>100	Photobacterium carnosum strain TMW 2.2021<br>Photobacterium phosphoreum strain NBRC 103031<br>Photobacterium aquimaris strain LC2-065	NR_156814.1<br>NR_114184.1<br>NR_041682.1	100<br>100<br>100	100<br>100<br>100	ON782614
PSD1-B	Stomach Digesta	551	Priestia megaterium strain Bme-BCI<br>Bacillus sp. (in: Bacteria) strain RBR43	ON544018.1<br>ON533692.1	100<br>100	100<br>100	Bacillus acanthi strain L28<br>Bacillus aryabhattai B8W22	MT516450.1<br>MK503498.1	100<br>100	100<br>100	ON782615

			Priestia megaterium strain LM-44	ON527364.1	100	100	Bacillus megaterium strain ATCC 14581	MK508856.1	100	100	
PSD2-B	Stomach Digesta	685	Shewanella putrefaciens strain S_T_TSA_82	JX860531.1	99.85	100	Shewanella putrefaciens strain ATCC 8071	NR_119141.1	99.27	100	ON782616
			Shewanella sp. strain X99	MF152119.1	99.85	100	Shewanella putrefaciens strain VTT E-95586	KU321263.1	99.27	100	
			Shewanella sp. strain X94	MF152114.1	99.85	100	Shewanella putrefaciens strain Hammer 95	NR_044863.1	99.27	100	
PSD4-B	Stomach Digesta	567	Shewanella sp. strain 7-55	MN945291.1	100	100	Shewanella colwelliana strain ATCC 39565	NR_043074.1	99.82	100	ON782617
			Shewanella colwelliana strain CeD-4	MN220615.1	100	100	Shewanella algidipiscicola strain NBRC 102032	NR_114023.1	99.29	100	
			Shewanella colwelliana strain CeD-3	MN220614.1	100	100	Shewanella algidipiscicola strain S13	NR_041297.1	99.29	100	
PSD5-B	Stomach Digesta	901	Vibrio anguillarum strain 2NS-PRS3-a2	MG264177.1	100	100	Vibrio qinghaiensis strain Q67	CP022741.1	99.0	100	ON782618
			Vibrio anguillarum strain VIB12	CP023310.1	100	100	Vibrio anguillarum strain DSM 21597	CP010084.1	99.0	100	
			Vibrio anguillarum strain VIB43	CP023054.1	100	100	Vibrio cortegadensis strain C 16.17	NR_148247.1	99.0	100	
PSD6-B	Stomach Digesta	909	Psychrobacter alimentarius strain JM48	MN758808.1	100	100	Psychrobacter aquaticus strain CMS 56	NR_042206.1	99.9	100	ON782619
			Psychrobacter alimentarius NGB-NV1	LC512308.1	100	100	Psychrobacter vallis strain CMS 39	NR_042205.1	99.9	100	
			Psychrobacter alimentarius NGB-ISM2	LC512280.1	100	100	Psychrobacter alimentarius strain JG-100	NR_025798.1	99.9	100	
PSD17-B	Stomach Digesta	570	Bacillus velezensis strain BN8.2	CP097288.2	100	100	Bacillus rugosus strain SPB7	MT554518.1	99.82	100	ON782620
			Bacillus sp. (in: Bacteria) strain ACZ06	ON533599.1	99.82	100	Bacillus subtilis subsp. subtilis str. 168	CP053102.1	99.82	100	
			Bacillus subtilis strain KA16	ON533518.1	99.82	100	Bacillus tequilensis strain KCTC 13622	MN543830.1	99.82	100	
MCC-PSD3-B	Stomach Digesta	873	Shewanella aestuarii strain Hal007	MT406388.1	100	100	Shewanella aestuarii strain JCM 17801	MT760375.1	99.20	100	ON782621
			Shewanella aestuarii strain Hal006	MT406387.1	100	100	Shewanella aestuarii strain SC18	NR_135728.1	99.08	100	
			Shewanella sp. AB411d	FR821223.1	100	100	Shewanella glacialimarina strain TZS-4	CP041216.1	98.28	100	
PI1-B	Intestine Epithelium	480	Bacillus sp. (in: Bacteria) strain A10	MT590643.1	100	100	Rossellomorea arthrocnemi strain EAR8	MZ416782.1	100	100	ON782622
			Bacillus vietnamensis strain A6	MT590649.1	100	100	Bacillus vietnamensis strain 15-1	MZ276305.1	100	100	
			Bacillus oryzaecorticis strain Y50-11	MT585201.1	100	100	Rossellomorea vietnamensis strain NBRC 101237	NR_113995.1	100	100	
PI2-B	Intestine Epithelium	869	Vibrio sp. VibC-Oc-072	KF577071.1	99.88	100	Vibrio gigantis strain LGP 13	NR_044079.1	99.54	100	ON782623
			Vibrio sp. VibC-Oc-070	KF577070.1	99.88	100	Vibrio crassostreae strain LGP 7	NR_044078.1	99.54	100	
			Vibrio sp. VibC-Oc-034	KF577048.1	99.88	100	Vibrio artabrorum strain VB 11.8	NR_116068.1	99.42	100	
PI4-B	Intestine Epithelium	401	Shewanella aestuarii strain Hal007	MT406388.1	100	100	Shewanella aestuarii strain SC18	NR_135728.1	99.5	99	ON782624
			Shewanella aestuarii strain Hal006	MT406387.1	100	100	Shewanella aestuarii strain JCM 17801	MT760375.1	99.5	99	
			Shewanella aestuarii strain Hal005	MT406386.1	100	100	Shewanella livingstonensis strain LMG 19866	CP034015.1	99.0	100	

PI8-B	Intestine Epithelium	495	Pseudoalteromonas sp. strain A8	MT591289.1	100	100	Pseudoalteromonas arctica A 37-1-2 chromosome II	CP011026.1	100	100	ON782625
			Pseudoalteromonas sp. strain WN8	MN889240.1	100	100	Pseudoalteromonas arctica A 37-1-2 chromosome I	CP011025.1	100	100	
			Pseudoalteromonas sp. strain WN6	MN889238.1	100	100	Pseudoalteromonas nigrifaciens strain KMM 661	CP011036.1	100	100	
PID1-B	Intestine Digesta	735	Vibrio anguillarum strain 2NS-PRS3-a2	MG264177.1	100	100	Vibrio cyclitrophicus strain LMG 21359	NR_115806.1	99.05	100	ON782626
			Vibrio anguillarum strain VIB12	CP023310.1	100	100	Vibrio mediterranei strain CECT 621	NR_117681.1	98.91	100	
			Vibrio anguillarum strain VIB43	CP023054.1	100	100	Vibrio pomeroyi strain CAIM 578	MT759944.1	98.91	100	
PID2-B	Intestine Digesta	810	Shewanella aestuarii strain Hal007	MT406388.1	100	100	Shewanella aestuarii strain JCM 17801	MT760375.1	99.4	100	ON782627
			Shewanella aestuarii strain Hal006	MT406387.1	100	100	Shewanella aestuarii strain SC18x	NR_135728.1	99.3	100	
			Shewanella sp. AB411d	FR821223.1	100	100	Shewanella gaetbuli strain TF-27	NR_135728.1	99.3	100	
PID3-B	Intestine Digesta	608	Photobacterium frigidiphilum strain QS227	MK439553.1	100	100	Photobacterium carnosum strain TMW 2.2021	NR_156814.1	100	100	ON782628
			Photobacterium sp. strain BOSW.4.10.32	MN960543.1	100	100	Photobacterium phosphoreum strain NBRC 103031	NR_114184.1	100	100	
			Photobacterium sp. strain TH13	MN049756.1	100	100	Photobacterium aquimaris strain LC2-065	NR_041682.1	100	100	
PID4-B	Intestine Digesta	588	Vibrio sp. strain GBPx3	MK560194.1	100	100	Vibrio qinghaiensis strain Q67	CP022741.1	99.8	100	ON782629
			Vibrio anguillarum strain 90-11-286	CP011460.1	100	100	Vibrio anguillarum strain DSM 21597	CP010084.1	99.2	100	
			Vibrio metschnikovii strain ST4	KJ486283.1	100	100	Vibrio metschnikovii strain NBRC 103153	NR_114220.1	99.2	100	
PID12-B	Intestine Digesta	749	Vibrio aestuarianus strain 15_075_1T2	MK307696.1	100	100	Vibrio aestuarianus subsp. cardii strain 12/122 3T3	MK307684.1	100	100	ON782630
			Vibrio aestuarianus strain 15_064_4T2	MK307695.1	100	100	Vibrio aestuarianus strain NBRC 15629	NR_113780.1	100	100	
			Vibrio aestuarianus strain 15_061_5T2	MK307693.1	100	100	Vibrio aestuarianus strain CAIM 592	MT759936.1	100	100	
PID21-B	Intestine Digesta	560	Bacillus pumilus strain BST3	ON533488.1	99.82	100	Bacillus aerophilus strain 28K	MN845151.1	99.82	100	ON782631
			Bacillus safensis strain BLXSC	CP034414.1	99.82	100	Bacillus altitudinis 41KF2b	MN840036.1	99.82	100	
			Bacillus stratosphericus strain NRSSBBSTRAT-1	ON520739.1	99.82	100	Bacillus aerius strain 24K	MN200939.1	99.82	100	
MCC-PID1-B	Intestine Digesta	814	Photobacterium phosphoreum strain 16LGm78	MK648254.1	100	100	Photobacterium carnosum strain TMW 2.2021	NR_156814.1	100	100	ON782632
			Photobacterium phosphoreum strain 15LGm78	MK641668.1	100	100	Photobacterium phosphoreum strain NBRC 103031	NR_114184.1	100	100	
			Photobacterium phosphoreum strain 9LMs78	MK641523.1	100	100	Photobacterium piscicola strain NCCB 100098	NR_125679.1	99.8	100	

W1-B	Water	519	Vibrio sp. strain CLE137<br>Vibrio sp. strain AEM-CIFE3 2012<br>Vibrio anguillarum K4 gene	MN931898.1<br>MK954131.1<br>LC475108.1	100<br>100<br>100	100<br>100<br>100	Vibrio qinghaiensis strain Q67<br>Vibrio anguillarum strain DSM 21597<br>Vibrio anguillarum strain NBRC 13266	CP022741.1<br>CP010084.1<br>NR_113609.1	100<br>100<br>100	100<br>100<br>100	ON782633
W2-B	Water	762	Psychrobacter sp. strain TH18<br>Psychrobacter sp. strain TH16<br>Psychrobacter sp. strain BH36	MN049761.1<br>MN049759.1<br>MN049674.1	99.87<br>99.87<br>99.87	100<br>100<br>100	Psychrobacter piscatorii strain T-3-2<br>Psychrobacter nivimaris strain 88/2-7<br>Psychrobacter muriicola strain 2pS	NR_112807.1<br>NR_028948.1<br>NR_114669.1	99.87<br>99.87<br>99.74	100<br>100<br>100	ON782634
W3-B	Water	672	Staphylococcus saprophyticus strain UTI-045<br>Staphylococcus edaphicus strain 2202<br>Staphylococcus edaphicus strain 674	CP054831.1<br>MT604700.1<br>MT585436.1	100<br>100<br>100	100<br>100<br>100	Staphylococcus succinus subsp. succinus strain DSM 14617<br>Staphylococcus succinus subsp. casei strain DSM 15096<br>Staphylococcus succinus subsp. succinus strain DSM 14617	MF678969.1<br>MF678968.1<br>MF678913.1	100<br>100<br>100	100<br>100<br>100	ON782635
W4-B	Water	786	Planococcus sp. IE3-4<br>Planococcus donghaensis strain DSM 22276<br>Planococcus donghaensis strain IARI-CDK 21	EF105393.1<br>CP016543.2<br>KT441070.1	100<br>99.8<br>99.6	100<br>100<br>100	Planococcus donghaensis strain DSM 22276<br>Planococcus donghaensis strain JH1<br>Planococcus donghaensis strain DSM 22276	CP016543.2<br>NR_044073.1<br>CP016544.1	99.8<br>99.6<br>99.5	100<br>100<br>100	ON782636
W5-B	Water	833	Bizionia hallyeonensis strain T-y7<br>Bizionia sp. M204<br>Bizionia sp. EM20	NR_109525.1<br>CP046242.1<br>GQ331110.1	100<br>99.9<br>99.8	100<br>100<br>100	Bizionia hallyeonensis strain T-y7<br>Bizionia echini strain KMM 6177<br>Bizionia algoritergicola strain APA-1	NR_109525.1<br>NR_116780.1<br>NR_043122.1	100<br>99.4<br>99.2	100<br>100<br>99	ON782637
W6-B	Water	880	Vibrio anguillarum strain MHK3<br>Vibrio anguillarum strain CNEVA NB11008<br>Vibrio anguillarum strain B26-1	CP022468.1<br>CP022103.1<br>KJ028214.1	100<br>100<br>100	100<br>100<br>100	Vibrio qinghaiensis strain Q67<br>Vibrio anguillarum strain DSM 21597<br>Vibrio cortegadensis strain C 16.17	CP022741.1<br>CP010084.1<br>NR_148247.1	98.9<br>98.9<br>98.9	100<br>100<br>100	ON782638
W10-B	Water	768	Arthrobacter echini isolate SW_2_206<br>Arthrobacter sp. strain AHE_PA_123<br>Arthrobacter sp. BB77	LR722945.1<br>MW580075.1<br>FR693359.1	100<br>100<br>100	99<br>99<br>99	Arthrobacter echini strain AM23<br>Arthrobacter ruber strain MDB1-42<br>Arthrobacter bussei strain KR32	NR_148833.1<br>MW227493.1<br>NR_170399.1	99.9<br>99.6<br>99.6	99<br>100<br>99	ON782639
W11-B	Water	698	Shewanella pealeana strain GSS<br>Shewanella pneumatophori isolate Q66<br>Shewanella sp. strain P8E-5	MN443612.1<br>LS482990.1<br>KX898880.1	99.86<br>99.71<br>99.71	100<br>100<br>100	Shewanella halifaxensis HAW-EB4<br>Shewanella pealeana strain ATCC 700345<br>Shewanella halifaxensis HAW-EB4	NR_074822.1<br>NR_074821.1<br>CP000931.1	99.71<br>99.71<br>99.71	100<br>100<br>100	ON782640
W12-B	Water	855	Rhodococcus sp. strain 23770<br>Rhodococcus sp. strain Bj13<br>Rhodococcus sp. strain ao1	MT357195.1<br>MT012186.1<br>MT012179.1	100<br>100<br>100	100<br>100<br>100	Rhodococcus fascians strain CF17<br>Rhodococcus fascians strain ATCC 12974<br>Rhodococcus cerastii strain C5	NR_037021.1<br>NR_119126.1<br>NR_117103.1	100<br>99.8<br>99.7	100<br>100<br>100	ON782641

W13-B	Water	565	Rhodococcus sp. strain 2b	MT484139.1	100	100	Rhodococcus fascians strain CF17	NR_037021.1	100	100	ON782642
			Rhodococcus sp. strain 23770	MT357195.1	100	100	Rhodococcus fascians strain ATCC 12974	NR_119126.1	99.7	100	
			Rhodococcus fascians strain VL_2051H	MT328751.1	100	100	Rhodococcus cerastii strain C5	NR_117103.1	99.5	100	
MCC-W1-B	Water	374	Shewanella kaireitica isolate R2A112_3_15	LR722823.1	100	100	Shewanella halifaxensis HAW-EB4	NR_074822.1	100	100	ON782643
			Shewanella sp. strain M20	MN049695.1	100	100	Shewanella pealeana strain ATCC 700345	NR_074821.1	100	100	
			Shewanella sp. strain Arc7-214	MN784326.1	100	100	Shewanella halifaxensis HAW-EB4	CP000931.1	100	100	
PG36-F	Gill	515	Inopinatum lactosum strain OUCMBIII101025	HQ914907.1	99.81	100	Inopinatum lactosum CCY 19-21-1 TYPE	NR_174911.1	100	100	ON791469
			Uncultured fungus clone ZBJ201205-13	KX514814.1	99.81	100	Inopinatum lactosum isolate D. Haelew. F-3088a	MW471139.1	100	100	
			Hyphozyma variabilis strain CNRMA12.453 isolate ISHAM-ITS_ID MITS2000	KP132305.1	99.81	100	Inopinatum lactosum isolate D. Haelew. F-3088b	MW471140.1	100	100	
PSD10-F	Stomach Digesta	440	Trichoderma harzianum isolate MT2	MT577837.1	100	100	Trichoderma lixii CBS 110080	NR_131264.1	99.5	100	ON791470
			Trichoderma harzianum isolate MT1	MT577649.1	100	100	Trichoderma asiaticum strain YMF1.00352	MH113930.1	99.3	99	
			Trichoderma lixii strain F-2	MT434003.1	100	100	Trichoderma atrobrunneum CBS 548.92	NR_137298.1	99.3	99	
PSD14-F	Stomach Digesta	423	Chromocleista sp. voucher research collection Farrer lab 27	MN644791.1	100	100	Thyridium curvatum CBS 490.82 TYPE	NR_132076.1	99.29	100	ON791471
			Paraconiothyrium cyclothyrioides voucher research collection Farrer lab 191	MN644673.1	100	100	Thyridium flavostromatum MAFF 247509 TYPE	NR_175611.1	99.05	100	
			Fusarium sporotrichioides voucher research collection Farrer lab 169	MN644658.1	100	100	Thyridium limonesiae CBS 146752 TYPE	NR_172355.1	98.35	100	
PI9-F	Intestine Epithelium	414	Aureobasidium pullulans isolate 21_3	MT363099.1	100	100	Aureobasidium pullulans strain CBS 584.75	KT693733.1	100	97	ON791472
			Uncultured fungus clone 4248_662	MT236651.1	100	100	Aureobasidium lini strain CBS 125.21	MH854694.1	98.8	99	
			Fungal sp. strain MP4S16	MT112993.1	100	100	Aureobasidium melanogenum CBS 105.22	NR_159598.1	98.1	100	
PID1-F	Intestine Digesta	573	Rhizopus microsporus strain 1M5	MT620751.1	100	100	Rhizopus azygosporus strain CBS 357.93	DQ119008.1	100	100	ON791473
			Rhizopus microsporus isolate 4	MT590587.1	100	100	Rhizopus microsporus var. chinensis strain CBS 631.82	MH861534.1	100	99	
			Rhizopus microsporus isolate GL58	MT590579.1	100	100	Rhizopus azygosporus strain CBS 357.93	MH862419.1	99.8	100	
PID20-F	Gut Digesta	437	Fusarium sporotrichioides voucher research collection Farrer lab 169	MN644658.1	100	100	Phialemoniopsis curvata strain: CBS 490.82	AB278180.1	99.1	100	ON791474
			Pleosporales sp. voucher research	MN644655.1	100	100	Thyridium curvatum CBS 490.82	NR_132076.1	99.1	97	

			collection Farrer lab 166								
			Phialemonium sp. 7/S/Pa/1/5/2	JX040769.1	100	100	Thyridium flavostromatum KT 3891	LC655959.1	98.8	97	
W9-F	Water	353	Penicillium antarcticum isolate CNUFC-RD48	MK390492.1	100	100	Penicillium antarcticum strain CBS 100492	MH862703.1	100	100	ON791475
			Penicillium sp. isolate CLE41	MN544011.1	100	100	Penicillium atrovenetum strain CBS 241.56	MH857605.1	99.7	100	
			Penicillium sp. isolate CLE177	MN544008.1	100	100	Penicillium atrovenetum NRRL 2571	NR_121255.1	99.7	100	

Table S2. Taxonomic identification of 66 microbial strains isolated from European plaice and the seawater reference to the highest possible level based on BLAST comparison to the NCBI nucleotide database (BLAST perfomed on June 13, 2022). Isolate codes: **PG**: Plaice Gut, **PS**: Plaice Stomach, **PSD**: Plaice Stomach Digesta, **PI**: Plaice Intestine Epithelium, **PID:** Plaice Intestine Digesta, **W**: Water reference, **MCC**: Isolation on Microchip using Fish Medium. Isolation medium abbreviations: **WSP**: modified Wickerham medium, **TSB**: Trypticase Soy Broth, **FM**: Fish Medium, **PDA**: Potato Dextrose Agar.

Strain ID	Source	Kingdom	Phylum	Class	Order	Family	Genus	Species	Isolation Medium
PG1-B	Gill	Bacteria	Proteobacteria	Gammaproteobacteria	Alteromonadales	Shewanellaceae	*Shewanella*	*baltica*	WSP
PG2-B	Gill	Bacteria	Proteobacteria	Gammaproteobacteria	Vibrionales	Vibrionaceae	*Vibrio*	*anguillarum*	WSP
PG4-B	Gill	Bacteria	Proteobacteria	Gammaproteobacteria	Vibrionales	Vibrionaceae	*Photobacterium*	sp.	WSP
PG5-B	Gill	Bacteria	Proteobacteria	Gammaproteobacteria	Pseudomonadales	Moraxellaceae	*Psychrobacter*	sp.	WSP
PG6-B	Gill	Bacteria	Proteobacteria	Gammaproteobacteria	Pseudomonadales	Moraxellaceae	*Psychrobacter*	sp.	TSB
PG8-B	Gill	Bacteria	Proteobacteria	Gammaproteobacteria	Pseudomonadales	Moraxellaceae	*Psychrobacter*	sp.	TSB
PG9-B	Gill	Bacteria	Proteobacteria	Gammaproteobacteria	Pseudomonadales	Moraxellaceae	*Psychrobacter*	sp.	TSB
PG10-B	Gill	Bacteria	Bacteroidetes	Flavobacteriia	Flavobacteriales	Weeksellaceae	*Chryseobacterium*	*carnis*	TSB
PG11-B	Gill	Bacteria	Actinomycetota	Actinobacteria	Micrococcales	Microbacteriaceae	*Microbacterium*	sp.	TSB
PG12-B	Gill	Bacteria	Proteobacteria	Gammaproteobacteria	Vibrionales	Vibrionaceae	*Vibrio*	*aestuarianus*	TSB
PG14-B	Gill	Bacteria	Proteobacteria	Gammaproteobacteria	Pseudomonadales	Moraxellaceae	*Psychrobacter*	*maritimus*	TSB
PG15-B	Gill	Bacteria	Proteobacteria	Gammaproteobacteria	Lysobacterales	Lysobacteraceae	*Stenotrophomonas*	sp.	PDA
PG16-B	Gill	Bacteria	Actinomycetota	Actinobacteria	Mycobacteriales	Nocardiaceae	*Rhodococcus*	sp.	PDA
PG17-B	Gill	Bacteria	Proteobacteria	Gammaproteobacteria	Pseudomonadales	Moraxellaceae	*Psychrobacter*	*maritimus*	WSP
PG18-B	Gill	Bacteria	Actinomycetota	Actinobacteria	Mycobacteriales	Nocardiaceae	*Rhodococcus*	sp.	WSP
PG19-B	Gill	Bacteria	Actinomycetota	Actinobacteria	Mycobacteriales	Nocardiaceae	*Rhodococcus*	sp.	WSP
PG20-B	Gill	Bacteria	Firmicutes	Bacilli	Caryophanales	Bacillaceae	*Bacillus*	sp.	TSB
PG25-B	Gill	Bacteria	Actinomycetota	Actinobacteria	Mycobacteriales	Nocardiaceae	*Rhodococcus*	sp.	TSB
PG26-B	Gill	Bacteria	Actinomycetota	Actinobacteria	Mycobacteriales	Nocardiaceae	*Rhodococcus*	sp.	TSB
PG31-B	Gill	Bacteria	Actinomycetota	Actinobacteria	Mycobacteriales	Nocardiaceae	*Rhodococcus*	sp.	TSB
PG32-B	Gill	Bacteria	Proteobacteria	Gammaproteobacteria	Pseudomonadales	Moraxellaceae	*Psychrobacter*	sp.	WSP

PG37-B	Gill	Bacteria	Proteobacteria	Gammaproteobacteria	Pseudomonadales	Moraxellaceae	*Psychrobacter*	sp.	TSB
PG38-B	Gill	Bacteria	Proteobacteria	Gammaproteobacteria	Pseudomonadales	Moraxellaceae	*Psychrobacter*	sp.	TSB
PG39-B	Gill	Bacteria	Proteobacteria	Alphaproteobacteria	Caulobacterales	Caulobacteraceae	*Brevundimonas*	*mediterranea*	WSP
PG42-B	Gill	Bacteria	Actinomycetota	Actinobacteria	Mycobacteriales	Dietziaceae	*Dietzia*	*alimentaria*	WSP
PG43-B	Gill	Bacteria	Actinomycetota	Actinobacteria	Micrococcales	Microbacteriaceae	*Microbacterium*	sp.	TSB
MCC-PG1-B	Gill	Bacteria	Proteobacteria	Gammaproteobacteria	Alteromonadales	Shewanellaceae	*Shewanella*	sp.	FM
MCC-PG2-B	Gill	Bacteria	Proteobacteria	Gammaproteobacteria	Pseudomonadales	Moraxellaceae	*Psychrobacter*	*maritimus*	FM
PS3-B	Stomach Epithelium	Bacteria	Proteobacteria	Gammaproteobacteria	Pseudomonadales	Moraxellaceae	*Psychrobacter*	sp.	TSB
MCC-PS2 -B	Stomach Epithelium	Bacteria	Proteobacteria	Gammaproteobacteria	Vibrionales	Vibrionaceae	*Photobacterium*	sp.	FM
PSD1-B	Stomach Digesta	Bacteria	Firmicutes	Bacilli	Caryophanales	Bacillaceae	*Bacillus*	sp.	TSB
PSD2-B	Stomach Digesta	Bacteria	Proteobacteria	Gammaproteobacteria	Alteromonadales	Shewanellaceae	*Shewanella*	sp.	TSB
PSD4-B	Stomach Digesta	Bacteria	Proteobacteria	Gammaproteobacteria	Alteromonadales	Shewanellaceae	*Shewanella*	*colwelliana*	TSB
PSD5-B	Stomach Digesta	Bacteria	Proteobacteria	Gammaproteobacteria	Vibrionales	Vibrionaceae	*Vibrio*	sp.	TSB
PSD6-B	Stomach Digesta	Bacteria	Proteobacteria	Gammaproteobacteria	Pseudomonadales	Moraxellaceae	*Psychrobacter*	*alimentarius*	TSB
PSD17-B	Stomach Digesta	Bacteria	Firmicutes	Bacilli	Caryophanales	Bacillaceae	*Bacillus*	*velezensis*	TSB
MCC-PSD3-B	Stomach Digesta	Bacteria	Proteobacteria	Gammaproteobacteria	Alteromonadales	Shewanellaceae	*Shewanella*	*aestuarii*	FM
PI1-B	Intestine Epithelium	Bacteria	Firmicutes	Bacilli	Caryophanales	Bacillaceae	*Bacillus*	sp.	TSB
PI2-B	Intestine Epithelium	Bacteria	Proteobacteria	Gammaproteobacteria	Vibrionales	Vibrionaceae	*Vibrio*	sp.	TSB
PI4-B	Intestine Epithelium	Bacteria	Proteobacteria	Gammaproteobacteria	Alteromonadales	Shewanellaceae	*Shewanella*	*aestuarii*	WSP
PI8-B	Intestine Epithelium	Bacteria	Proteobacteria	Gammaproteobacteria	Alteromonadales	Pseudoalteromonadaceae	*Pseudoalteromonas*	sp.	WSP

PID1-B	Intestine Digesta	Bacteria	Proteobacteria	Gammaproteobacteria	Vibrionales	Vibrionaceae	*Vibrio*	*anguillarum*	TSB
PID2-B	Intestine Digesta	Bacteria	Proteobacteria	Gammaproteobacteria	Alteromonadales	Shewanellaceae	*Shewanella*	*aestuarii*	TSB
PID3-B	Intestine Digesta	Bacteria	Proteobacteria	Gammaproteobacteria	Vibrionales	Vibrionaceae	*Photobacterium*	sp.	TSB
PID4-B	Intestine Digesta	Bacteria	Proteobacteria	Gammaproteobacteria	Vibrionales	Vibrionaceae	*Vibrio*	sp.	WSP
PID12-B	Intestine Digesta	Bacteria	Proteobacteria	Gammaproteobacteria	Vibrionales	Vibrionaceae	*Vibrio*	*aestuarianus*	TSB
PID21-B	Intestine Digesta	Bacteria	Firmicutes	Bacilli	Caryophanales	Bacillaceae	*Bacillus*	sp.	TSB
MCC-PID1-B	Intestine Digesta	Bacteria	Proteobacteria	Gammaproteobacteria	Vibrionales	Vibrionaceae	*Photobacterium*	sp.	FM
W1-B	Water	Bacteria	Proteobacteria	Gammaproteobacteria	Vibrionales	Vibrionaceae	*Vibrio*	sp.	TSB
W2-B	Water	Bacteria	Proteobacteria	Gammaproteobacteria	Pseudomonadales	Moraxellaceae	*Psychrobacter*	sp.	TSB
W3-B	Water	Bacteria	Firmicutes	Bacilli	Caryophanales	Staphylococcaceae	*Staphylococcus*	sp.	TSB
W4-B	Water	Bacteria	Firmicutes	Bacilli	Caryophanales	Caryophanaceae	*Planococcus*	*donghaensis*	TSB
W5-B	Water	Bacteria	Bacteroidetes	Flavobacteriia	Flavobacteriales	Flavobacteriaceae	*Bizionia*	*hallyeonensis*	TSB
W6-B	Water	Bacteria	Proteobacteria	Gammaproteobacteria	Vibrionales	Vibrionaceae	*Vibrio*	sp.	WSP
W10-B	Water	Bacteria	Actinomycetota	Actinobacteria	Micrococcales	Micrococcaceae	*Arthrobacter*	*echini*	TSB
W11-B	Water	Bacteria	Proteobacteria	Gammaproteobacteria	Alteromonadales	Shewanellaceae	*Shewanella*	*pealeana*	TSB
W12-B	Water	Bacteria	Actinomycetota	Actinobacteria	Mycobacteriales	Nocardiaceae	*Rhodococcus*	sp.	PDA
W13-B	Water	Bacteria	Actinomycetota	Actinobacteria	Mycobacteriales	Nocardiaceae	*Rhodococcus*	*fascians*	PDA
MCC-W1-B	Water	Bacteria	Proteobacteria	Gammaproteobacteria	Alteromonadales	Shewanellaceae	*Shewanella*	sp.	FM
PG36-F	Gill	Fungi	Ascomycota	Leotiomycetes	incertae sedis	incertae sedis	incertae sedis	n/a	WSP
PSD10-F	Stomach Digesta	Fungi	Ascomycota	Sordariomycetes	Hypocreales	Hypocreaceae	*Trichoderma*	sp.	WSP
PSD14-F	Stomach Digesta	Fungi	Ascomycota	Sordariomycetes	incertae sedis	incertae sedis	incertae sedis	n/a	WSP
PI9-F	Intestine Epithelium	Fungi	Ascomycota	Dothideomycetes	Dothideales	Saccotheciaceae	*Aureobasidium*	*pullulans*	PDA

PID1-F	Intestine Digesta	Fungi	Mucoromycota	Mucoromycetes	Mucorales	Mucoraceae	*Rhizopus*	sp.	PDA
PID20-F	Intestine Digesta	Fungi	Ascomycota	Sordariomycetes	incertae sedis	incertae sedis	incertae sedis	n/a	PDA
W9-F	Water	Fungi	Ascomycota	Eurotiomycetes	Eurotiales	Aspergillaceae	*Penicillium*	*antarcticum*	PDA

Table S3. Biological activity of all 23 selected plaice-associated microorganisms. n.g. No growth. Vi: *V. ichthyoenteri.* Lg: *Lactococcus garvieae*, MRSA: Methillicin-resistant *Staphylococcus aureus*, Ef: *Enterococcus faecium*. Positive controls: Chloramphenicol (Vi and MRSA), Ampicillin (Lg and Ef).

Code	Taxonomical ID	Origin	Medium - regime	*Vi*	*Lg*	MRSA	*Ef*
PG1-B	*Shewanella baltica*	Gill	MA solid	> 100	> 100	20.2	26.4
			MB liquid	> 100	41.7	18.8	8.8
PG10-B	*Chryseobacterium carnis*	Gill	MA solid	18.6	> 100	39.0	41.9
			MB liquid	n.c.	> 100	n.c.	51.0
PG11-B	*Microbacterium* sp.	Gill	MA solid	> 100	> 100	45.9	86.6
			MB liquid	5.7	> 100	> 100	> 100
PG12-B	*Vibrio aestuarianus*	Gill	MA solid	17.2	27.8	19.1	34.4
			MB liquid	43.9	> 100	61.0	43.4
PG19-B	*Rhodococcus* sp.	Gill	MA solid	> 100	> 100	> 100	> 100
			MB liquid	> 100	> 100	> 100	> 100
PG20-B	*Bacillus sp.*	Gill	MA solid	> 100	> 100	> 100	> 100
			MB liquid	> 100	> 100	> 100	> 100
PG25-B	*Rhodococcus sp*	Gill	MA solid	> 100	> 100	> 100	> 100
			MB liquid	> 100	> 100	> 100	> 100
PG26-B	*Rhodococcus sp*	Gill	MA solid	> 100	> 100	> 100	> 100
			MB liquid	> 100	> 100	> 100	> 100
PG31-B	*Rhodococcus sp*	Gill	MA solid	> 100	> 100	> 100	> 100
			MB liquid	> 100	> 100	> 100	> 100
PG8-B	*Psychrobacter sp.*	Gill	MA solid	> 100	> 100	> 100	> 100
			MB liquid	> 100	> 100	> 100	> 100
PG42-B	*Dietzia alimentaria*	Gill	MA solid	> 100	> 100	> 100	> 100
			MB liquid	> 100	> 100	> 100	> 100
PG39-B	*Brevundimonas mediterranea*	Gill	MA solid	> 100	> 100	> 100	> 100
			MB liquid	> 100	> 100	> 100	> 100
PS3-B	*Psychrobacter* sp.	Gut (Stomach) Epithelium)	MA solid	> 100	> 100	23.3	28.9
			MB liquid	> 100	> 100	11.4	7.5
PSD1-B	*Bacillus sp.*	Gut (Stomach) Digesta	MA solid	> 100	> 100	> 100	> 100
			MB liquid	> 100	> 100	> 100	> 100
PSD4-B	*Shewanella colwelliana*		MA solid	> 100	25.4	29.1	30.8

		Gut (Stomach) Digesta	MB liquid	n.g.	n.g.	n.g.	n.g.
PI1-B	*Bacillus* sp.	Gut (Intestine) Epithelium	MA solid	> 100	14.7	18.5	15.0
			MB liquid	> 100	54.2	22.1	11.6
PI2-B	*Vibrio* sp.	Gut (Intestine) Epithelium	MA solid	> 100	14.1	15.8	17.5
			MB liquid	> 100	> 100	36.4	9.0
PI8-B	*Pseudoalteromonas* sp.	Gut (Intestine) Epithelium	MA solid	> 100	28.3	21.2	50.4
			MB liquid	> 100	36.6	8.1	8.2
PID2-B	*Shewanella aestuarii*	Gut (Intestine)Digesta	MA solid	n.g.	n.g	n.g	n.g.
			MB liquid	> 100	> 100	6.8	4.9
PID21-B	*Bacillus* sp.	Gut (Intestine) Digesta	MA solid	4.7	> 100	10.2	> 100
			MB liquid	28.4	> 100	5.9	> 100
MCC-PID1-B	*Photobacterium* sp.	Gut (Intestine) Digesta	MA solid	> 100	40.2	35.0	69.0
			MB liquid	n.g.	n.g.	n.g.	n.g.
PI9-F	*Aureobasidium pullulans*	Gut (Intestine) Epithelium	PDA solid	> 100	22.6	40.8	30.7
			PDA liquid	> 100	> 100	8.9	3.3
PG36-F	*Leotiomycetes incertae sedis*	Gill	PDA solid	>100	>100	> 100	> 100
			PDA liquid	>100	>100	> 100	> 100
	Positive control	-	-	0.4	0.5	1.5	0.2

Table S4. Putative annotation of metabolites detected in *Bacillus* sp. PID21-B. t_R: Retention time

ID	Exp. m/z	t_R (min)	Δ ppm	Putative molecular formula	Ionization	MS/MS fragmentation pattern	Putatively annotated compound(s)	Chemical family	Biological Source	Reported bioactivity	Structures	Reference(s)
P21-1	328.2858	10.9192	1.5	$C_{19}H_{37}NO_3$	$[M+H]^+$	217.8850, 95.0867, 245.8821, 123.1166, 76.0407	Alanine; *N*-pentadecanoyl, Me ester	N-acetyl amines	many microorganisms		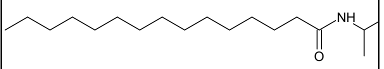	[1]
P21-2	412.1368	6.323	0.2	$C_{20}H_{23}NO_7$	$[M+Na]^+$	272.1258, 394.1268	AI-77-F	Isocoumarin	*Bacillus pumilus*	antibacterial, antiulcer	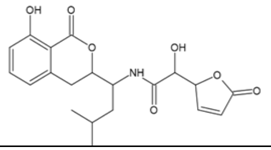	[1]
P21-3	323.195	6.5926	1.5	$C_{15}H_{28}N_2O_4$	$[M+Na]^+$		Lipoamide A	N-acyl amine	*Bacillus pumilus*	antibacterial	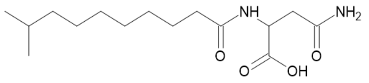	[2]
P21-4	955.5202	7.9466	NA			489.2563, 276.1196, 258.1086, 491.2620, 375.1877					Unidentified	
P21-5	337.2107	7.1797	1.2	$C_{16}H_{30}N_2O_4$	$[M+Na]^+$	133.06, 200.20, 298.20, 109.1	Lipoamide B	N-acyl amine	*Bacillus pumilus*	not reported	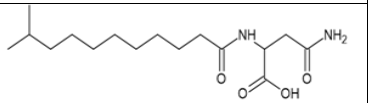	[2]
P21-6	439.208	4.7314	NA			232.1344, 250.1449, 215.1078, 176.0713, 149.0901, 422.1825,					Unidentified	
P21-7	312.2177	7.7286	1.9	$C_{17}H_{29}NO_4$	$[M+H]^+$	214.2179, 88.0409	Antibiotic Y 03559J-A	Fatty acid	*Micromonospora* sp.	Gram positiv	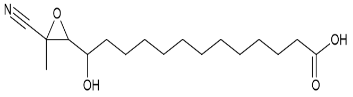	[3]
P21-8	467.2731	7.9527	-0.5	$C_{24}H_{38}N_2O_7$	$[M+H]^+$	326.8519, 257.0879	Farneside A	Terpenoid (mixed)	Marine *Streptomyces*	Antimalarial	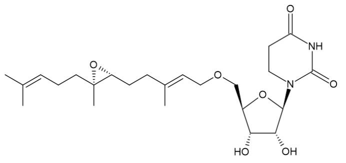	[4]

P21-9	911.5562	7.9466	NA			467.2738, 354.1901, 254.1378, 155.0683					Unidentified	
P21-10	445.2912	7.9527	NA			186.1484, 72.0834, 214.1451, 314.1936,					Unidentified	
P21-11	889.5519	11.3895	NA								Unidentified	
P21-12	326.2699	10.3931	1.5	$C_{19}H_{35}NO_3$	$[M+H]^+$	308.2589	Fluvirucin C_2	Macrolide	Marine Actinomycete		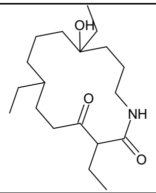	[5]
P21-13	663.3562	7.8588	NA			365.1068, 501.3032					Unidentified	
P21-14	390.155	6.323	0.1	$C_{20}H_{23}NO_7$	$[M+H]^+$	215.1065, 232.1329, 159.0439, 250.1431, 123.0082, 149.0592, 176.0707, 197.0955	AI-77-F	Isocoumarin	*Bacillus pumilus*		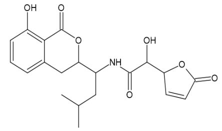	[1]
P21-15	301.2132	6.5926	2.5	$C_{15}H_{28}N2O_4$	$[M+H]^+$	133.0613, 186.1860, 284.1867, 151.14, 95.0860, 116.0345	Lipoamide A	N-acyl amine	*Bacillus pumilus*		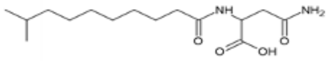	[2]
P21-16	351.2258	7.7286	0.6	$C_{17}H_{32}N_2O_4$	$[M+Na]^+$	155.0427, 254.8679,	Lipoamide C	N-acyl amine	*Bacillus pumilus*		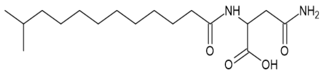	[2]
P21-17	407.1816	4.5482	-0.3	$C_{20}H_{26}N_2O_7$	$[M+H]^+$	215.1066, 232.1334, 159.0539, 250.1443, 274.0711, 390.1536, 175.0388	Amicoumacin C	Isocoumarin	*Bacillus pumilus*	antibacterial, antiulcer	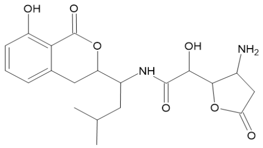	[6]
P21-18	315.2288	7.1797	0	$C_{16}H_{30}N_2O_4$	$[M+H]^+$	133.0616, 200.2015, 298.2014, 109.1020, 85.0550, 165.1649	Lipoamide B	N-acyl amines	*Bacillus pumilus*		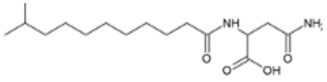	[2]

P21-19	479.3458	10.3625	-0.2	$C_{25}H_{48}N_2O_5$	$[M+Na]^+$	355.261	Fluvirucin B	Polyketides	*Actinomyces* sp.	not reported	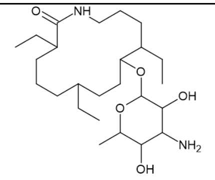	[5]
P21-20	300.2546	9.8264	NA			95.0864, 109.1016, 123.1177, 76.0404, 137.1322, 81.0698					Unidentified	
P21-21	314.2702	10.4201	2.2	$C_{18}H_{35}NO_3$	$[M+H]^+$	95.0860, 109.1026, 215.8649, 123.1157	Alanine-N-Tetradecanoylmethyl ester	N-acetyl amines	many microorganisms			[7]
P21-22	424.2078	4.2692	0.5	$C_{20}H_{29}N_3O_7$	$[M+H]^+$	232.1339, 250.1447, 215.1075, 274.0721, 407.1824, 390.1551, 159.0445, 175.0395, 330.1344	Amicoumacin A		*Bacillus pumilus*	Antimicrobial, antiulcer, antiinflamma-tory, anticholestero-lemic	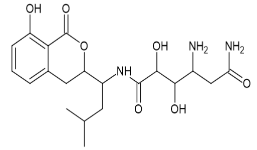	[6]

Table S5. Putative annotation of metabolites detected in *Bacillus* sp. PI1-B. t_R: Retention time

ID	Exp. m/z	t_R (min)	Δ ppm	Putative molecular formula	Ionization	MS/MS fragmentation pattern	Putatively annotated compound(s)	Chemical family	Biological Source	Reported bioactivity	Structures	Reference(s)
PI1-1	417.1489	6.0213	0.2	$C_{15}H_{26}N2O_{10}$	$[M+Na]^+$	266.1366, 139.0844,217.0448, 337.1729, 329.0967, 307.1156	Enkastine; Enkastine 2	Glycopeptide	*Streptomyces albus*	inhibitor of the endopeptidase	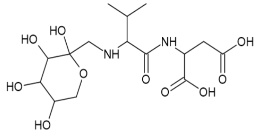	[8]
PI1-2	352.2458	5.1465	NA			254.8668					Unidentified	
PI1-3	331.169	4.3803	NA			261.1249, 159.0585, 142.0859, 184.0963, 243.1148, 297.0343, 99.0562					Unidentified	
PI1-4	268.2636	10.787	1.2	$C_{17}H_{33}NO$	$[M+H]^+$	135.1166, 149.1318, 233.2263, 121.1011					Unidentified	
PI1-5	465.1491	6.6292	NA			329.0951, 139.0841, 314.1358, 307.1119, 273.0329, 157.0422					Unidentified	

Table S6. Putative annotation of metabolites detected in *Microbacterium* sp. PG11-B

ID	Exp. m/z	t_R (min)	Δ ppm	Putative molecular formula	Ionization	MS/MS fragmentation pattern	Putatively annotated compound(s)	Chemical family	Biological Source	Reported bioactivity	Structures	Reference(s)
PG11-1	716.5224	11.339	NA			549.4882, 280.2655, 237.2218					Unidentified	

Table S7. Putative annotation of metabolites detected in *Chryseobacterium carnis* PG10-B

ID	Exp. m/z	t_R (min)	Δ ppm	Putative molecular formula	Ionization	MS/MS fragmentation pattern	Putatively annotated compound(s)	Chemical family	Biological Source	Reported bioactivity	Structures	Reference(s)
PG10-1	641.7462	10.9422	NA			115.08,366.30, 347.30, 70.06, 319.30					Unidentified	
PG10-2	627.7277	10.7071	NA			115.08, 352.28, 333.28, 305.29, 70.06					Unidentified	
PG10-3	627.5311	10.7096	NA			115.08, 352.28, 333.28, 305.29, 70.07					Unidentified	
PG10-4	325.2734	8.3097	-4.6	$C_{18}H_{38}O_3$	$[M+Na]^+$	215.8904, 189.8653, 95.0873, 243.8862	3-hydroxy-16-methylheptadecanoic acid	Fatty acid	*Bacillus* sp.			[9]
PG10-5	490.1445	5.1638	4.3	$C_{17}H_{23}N_5O_{12}$	$[M+H]^+$	301.11, 360.08, 416.07, 171.05, 472.13	Polyoxin O	nucleoside antibiotic	*Streptomyces piomogenus*	antifungal	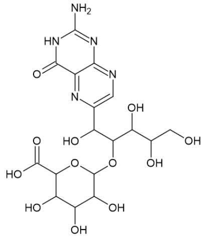	[10]
PG10-6	419.3118	6.2497	NA			96.04, 353.27, 113.07, 126.05, 240.23, 335.26					Unidentified	
PG10-7	433.3271	6.7091	NA			96.04, 367.29, 126.05, 144.06, 254.24, 113.07					Unidentified	
PG10-8	433.3278	6.6721	NA								Unidentified	
PG10-9	405.2955	5.8762	NA			96.04, 339.26, 226.21, 126.05, 322.24					Unidentified	
PG10-10	613.5155	10.4721	NA			115.08, 338.26, 352.28, 319.27,					Unidentified	
PG10-11	312.1107	5.4846	NA			156.88, 254.92, 230.88, 210.93					Unidentified	

PG10-12	649.5128	10.7105	NA			137.0691, 391.2927, 373.2812, 535.4332, 649.5107					Unidentified	
PG10-13	663.5285	10.9442	NA			137.0687, 405.3062, 387.2975, 549.4487, 646.5020					Unidentified	
PG10-14	639.5308	10.5996	NA								Unidentified	

Table S8. Putative annotation of metabolites detected in *Shewanella baltica* PG1-B

ID	Exp. m/z	t_R (min)	Δ ppm	Putative molecular formula	Ionization	MS/MS fragmentation pattern	Putatively annotated compound(s)	Chemical family	Biological Source	Reported bioactivity	Structures	Reference(s)
PG1-1	377.2681	10.0943	NA			285.22, 145.10, 267.21, 173.13, 187.14, 201.16, 131.08, 159.11					Unidentified	
PG1-2	781.5613	8.375	$[2M+H]^+$	$C_{24}H_{38}O_4$		355.2639, 337.2531, 319.2430, 213.1642, 159.1178, 133.1078	(3α,5β)-3-Hydroxy-6-oxocholan-24-oic acid	Terpenoid			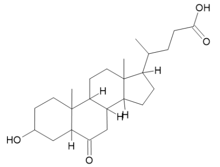	https://www.chemspider.com/Chemical-Structure.144621.html
PG1-3	753.5283	10.3714	NA			735.5281, 735.5144, 559.4728, 407.3122					Unidentified	
PG1-4	303.2319	10.666	NA			119.0858, 161.1331, 133.1014, 105.0713					Unidentified	
PG1-5	835.5328	6.8779	$[2M+Na]^+$	$C_{24}H_{38}O_5$		429.2616	(3β,5β,7β)-3,7-Dihydroxy-12-oxocholan-24-oic acid	Terpenoid			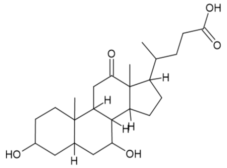	
PG1-6	614.4932	9.3475	NA			150.0546					Unidentified	
PG1-7	485.2232	8.375	NA			315.2597, 215.1812					Unidentified	
PG1-8	771.5393	8.8353	NA			753.5294, 577.4780					Unidentified	

PG1-9	337.2535	8.375	NA			319.2427, 213.1646, 267.2116, 159.1172, 279.2107					Unidentified	
PG1-10	775.5491	10.9359	NA			757.5405					Unidentified	
PG1-11	609.5212	10.8623	NA			115.0873, 350.2710, 367.2965, 303.2797					Unidentified	
PG1-12	614.4937	7.979	NA			150.0551					Unidentified	
PG1-13	813.5508	6.8779	NA			371.2585, 355.2639, 353.2474, 335.2375, 319.2418, 245.1538, 199.1478, 159.1173					Unidentified	

Table S9. Putative annotation of metabolites detected in *Shewanella aestuarii* PID2-B

ID	Exp. m/z	t_R (min)	Δ ppm	Putative molecular formula	Ionization	MS/MS fragmentation pattern	Putatively annotated compound(s)	Chemical family	Biological Source	Reported bioactivity	Structures	Reference(s)
PID2-1	263.2381	11.3215		$C_{18}H_{32}O_2$	$[M+H-H_2O]^+$	245.2276, 95.0856, 109.1010, 133.1012, 147.1177	10E,Z12-CLA	fatty acid			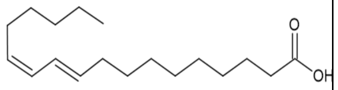	
PID2-2	499.3762	11.0524	NA		$[M+Na]^+$	332.7257, 481.3620					Unidentified	
					$[M+H]^+$						Unidentified	
PID2-3	642.4704	11.0127	NA			483.3797, 182.0799					Unidentified	
PID2-4	501.3918	10.5022	NA			483.3795, 194.1304					Unidentified	
PID2-5	517.3865	8.6097	NA			499.3706					Unidentified	
PID2-6	517.3868	6.8578	NA			499.3751, 277.1782					Unidentified	
PID2-7	643.4185	8.8808	0.6	$C_{36}H_{60}O_8$	$[M+Na]^+$	203.0506	21-deoxy-bafilomycin A2		*Streptomyces* sp.	Antibiotic	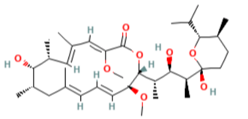	[11]
PID2-8	600.4603	8.1608	NA			483.38					Unidentified	
PID2-9	563.3495	10.9754				433.2869, 367.8052, 184.8905					Unidentified	
PID2-10	281.2124	9.1462	NA		$[M+H]^+$	168.079					Unidentified	
PID2-11	239.1655	8.5331	0.4	C14H22O3	$[M+H]^+$	211.1695, 126.0319	Violapyrone C	α-pyrone	*Streptomyces violascens*		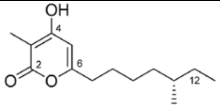	[12]
PID2-12	578.4783	8.1608	0.3	$C_{36}H_{63}NO_5$	$[M+H]^+$	118.0867, 72.0816, 135.1178	35-Amino-3-methyl-11-bacteriohopene-30,31,32,33,34-pentol	Terpenoids	*Methylocaldum szegediense*		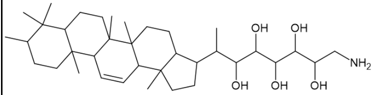	[13]
PID2-13	515.3712	7.4033	NA		$[M+H]^+$	497.3605, 275.1620					Unidentified	

Table S10. Putative annotation of metabolites detected in *Shewanella colwelliana* PSD4-B

ID	Exp. m/z	t_R (min)	Δ ppm	Putative molecular formula	Ionization	MS/MS fragmentation pattern	Putatively annotated compound(s)	Chemical family	Biological Source	Reported bioactivity	Structures	Reference(s)
PSD4-1	554.330261	8.5863	NA			226.0707, 351.2501, 434.2899					Unidentified	
PSD4-2	329.270187	10.27301 67	NA			219.2118, 135.1173, 121.1019, 311.2581, 107.0856, 95.0866					Unidentified	
PSD4-3	513.304001	8.728766 67	NA			513.3036					Unidentified	
PSD4-4	303.2327	10.7001	1.3		$[M+H]^+$	133.1015, 161.1332, 221.1540, 175.1487, 257.1912, 119,0855, 207.1383, 233.1543	$C_{20}H_{30}O_2$ (270 hits)				Unidentified	

Table S11. Putative annotation of metabolites detected in *Psychrobacter* sp. PS3-B

ID	Exp. m/z	t_R (min)	Δ ppm	Putative molecular formula	Ionization	MS/MS fragmentation pattern	Putatively annotated compound(s)	Chemical family	Biological Source	Reported bioactivity	Structures	Reference(s)
PS3-1	448.306	6.039		$C_{26}H_{43}NO_6$	$[M-H_2O+H]^+$		Glycocholic acid<br><br>3,7,12-trihydroxy-10,13-dimethylhexadecahydro-1H-cyclopenta[a]phenanthren-17-yl)pentanoyl)glycine	Bile acid			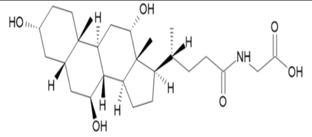	https://www.chemspider.com/Chemical-Structure.9734.html
PS3-2	430.2956	6.039		$C_{26}H_{43}NO_6$	$[M-2H_2O+H]^+$		Glycocholic acid<br><br>3,7,12-trihydroxy-10,13-dimethylhexadecahydro-1H-cyclopenta[a]phenanthren-17-yl)pentanoyl)glycine	Bile acid			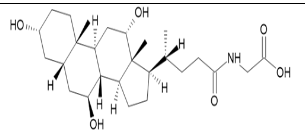	https://www.chemspider.com/Chemical-Structure.9734.html
PS3-3	466.3165	6.039		$C_{26}H_{43}NO_6$	$[M+H]^+$		Glycocholic acid<br><br>3,7,12-trihydroxy-10,13-dimethylhexadecahydro-1H-cyclopenta[a]phenanthren-17-yl)pentanoyl)glycine	Bile acid			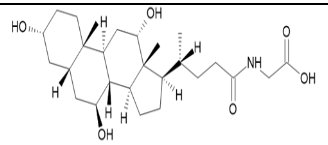	https://www.chemspider.com/Chemical-Structure.9734.html
PS3-4	312.2906	10.3647	NA		294.2798, 135.1184, 215.8662, 149.1329, 177.1669, 95.0867		No hit				Unidentified	
PS3-5	272.2595	9.313	2.2	$C_{16}H_{34}NO_2$	255.2331, 230.2488, 95.0865		C16-Sphingosine<br><br>2-Amino-4-hexadecane-1,3-diol	Aminolipid	constitute of various sphingolipids		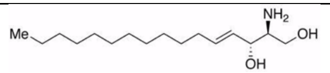	[14]

Table S12. Putative annotation of metabolites detected in *Vibrio aestuarianus* PG12-B

ID	Exp. m/z	t_R (min)	Δ ppm	Putative molecular formula	Ionization	MS/MS fragmentation pattern	Putatively annotated compound(s)	Chemical family	Biological Source	Reported bioactivity	Structures	Reference(s)
PG12-1	144.045	6.1096	NA			103.9569, 116.0502					Unidentified	
PG12-2	419.2878	9.5502	NA								Unidentified	
PG12-3	405.272	8.8097	NA			151.0477, 388.2445, 170.0609					Unidentified	
PG12-4	366.2639	8.8948	NA			84.0462, 102.0553, 256.2645, 235.8719					Unidentified	
PG12-5	368.2791	9.6612	-2.7	$C_{21}H_{37}NO_4$	$[M+H]^+$	84.0463, 102.0559	Tumonoic acid A; Et ester	polyketide	*L. majuscula* and S. *calcicola*	anti-inflammatory	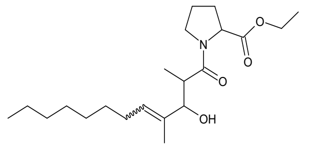	[15]
PG12-6	339.2639	9.5781	NA								Unidentified	
PG12-7	383.2792	10.3954	-1.8	$C_{22}H_{38}O_5$	$[M+H]^+$	291.2315, 165.1270, 235.1685, 109.0657	4-Hydroxy-5-(hydroxymethyl)-3-(14-methyl-hexadecanoyl)-2(5H)-furanone	Polyketide	*Streptomyces* sp	protease inhibitors	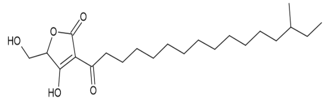	[16]
PG12-8	520.3036	10.4158	NA								Unidentified	
PG12-9	367.2956	10.7281	NA			129.0660, 84.0466, 256.2635, 95.0837					Unidentified	
PG12-10	364.2615	7.9426	NA			98.9884, 142.0260, 235.8777					Unidentified	
PG12-11	365.2799	9.9365	NA			129.0660, 84.0463, 121.1017, 135.1168, 95.0867, 149.1317, 219.2099						

Table S13. Putative annotation of metabolites detected in *Vibrio* sp. PI2-B

ID	Exp. m/z	t_R (min)	Δ ppm	Putative molecular formula	Ionization	MS/MS fragmentation pattern	Putatively annotated compound(s)	Chemical family	Biological Source	Reported bioactivity	Structures	Reference(s)
1	286.0937	3.2199	NA			258.0984, 241.0716, 216.0765, 269.0666, 134.0599					Unidentified	
P2- 2	397.2693	9.2215	NA			144.0201, 161.0556, 88.0406, 115.0515					Unidentified	
P2-3	579.3203	8.5626	NA			269.0685, 171.0920, 311.2577, 144.0807, 189.1022					Unidentified	
P2-4	331.0917	4.1921	NA			201.8844, 157.8972, 252.8306					Unidentified	
P2-5	380.1368	5.5337	-2.4	$C_{22}H_{19}N_3O_2$	$[M+Na]^+$	182.0490, 250.1128	Marinacarboline C	alkaloid	*Marinactinospora thermotolerans*	antiplasmodial	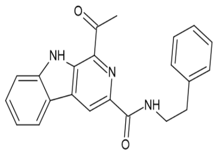	[17]

Table S14. Putative annotation of metabolites detected in *Pseudoalteromonas* sp. PI8-B

ID	Exp. m/z	t_R (min)	Δ ppm	Putative molecular formula	Ionization	MS/MS fragmentation pattern	Putatively annotated compound(s)	Chemical family	Biological Source	Reported bioactivity	Structures	Reference(s)
P8-1	699.5643	11.4854	NA			585.4852, 417.3098, 137.0691, 399.2975, 681.5519					Unidentified	
	673.549	11.4364	NA			137.06, 656.52, 559.47, 115.08, 378.29, 391.29					Unidentified	
P8-3	327.2161	8.4704	-1.8	$C_{18}H_{30}O_5$	$[M+H]^+$	191.14, 235.16, 137.09, 207.17, 137.09	Albocycline B	Macrolide	*Streptomyces* sp	antibacterial	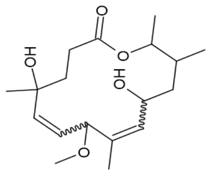	[18]
P8-4	677.5831	11.4854	NA			115.08, 378.30, 395.32, 359.30, 331.31, 263.23, 159.07					Unidentified	

Table S15. Putative annotation of metabolites detected in *Aureobasidium pullulans* PI9-F

ID	Exp. m/z	t_R (min)	Δ ppm	Putative molecular formula	Ionization	MS/MS fragmentation pattern	Putatively annotated compound(s)	Chemical family	Biological Source	Reported bioactivity	Structures	Reference(s)
PI9-1	577.3199	6.3572		$C_{26}H_{50}O_{12}$	[M+Na]+	373.1839, 227.1265, 275.0748, 109.1014, 559.3016	3-hydroxy-1-oxo-1-(2,3,4,5,6-pentahydroxyhexoxy) decan-5-yl] 3,5-dihydroxydecanoate	Lipid			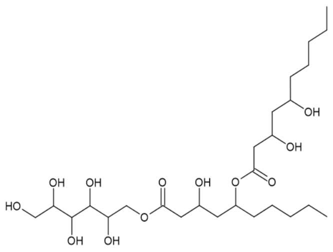	PubChem [Internet]. Bethesda (MD): National Library of Medicine (US), National Center for Biotechnology Information; 2004-. PubChem Compound Summary for CID 23843944; [cited 2022 July 19]. Available from: https://pubchem.ncbi.nlm.nih.gov/compound/23843944
PI9-2	745.5097	10.4858		$C_{40}H_{74}O_{13}$	[M-H_2O+H]+	109.1019, 127.1126, 301.2123	5-[5-[5-(3,5-dihydroxydecanoyloxy)-3-hydroxydecanoyl]oxy-3-hydroxydecanoyl]oxy-3-hydroxydecanoic acid	Lipid			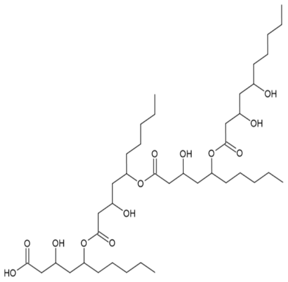	

PI9-3	745.5096	10.1982		$C_{40}H_{74}O_{13}$	[M-H_2O+H]+	109.1017, 127.1125, 133.1017, 151.1118, 169.1230	5-[5-[5-(3,5-dihydroxydecanoylox)-3-hydroxydecanoyl]oxy-3-hydroxydecanoyl]oxy-3-hydroxydecanoic acid	Lipid				
PI9-4	705.4425	8.1025		$C_{36}H_{68}O_{15}$	[M-2H_2O+H]$^+$	109.1017, 151.1113, 129.0556, 165.0772, 414.2536, 499.2908	[3-hydroxy-1-[3-hydroxy-1-oxo-1-(2,3,4,5,6-pentahydroxyhexoxy)decan-5-yl]oxy-1-oxodecan-5-yl] 3,5-dihydroxydecanoate<br><br>Liamocin A1	Lipid	*Aureobasidium pullulans*	antibacterial	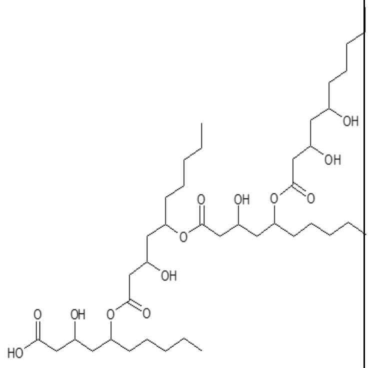	[19]
PI9-5	763.4455	8.1025		$C_{36}H_{68}O_{15}$	[M+Na]+	373.1857, 413.2517, 559.3098, 227.1268, 209.1161	3-hydroxy-1-[3-hydroxy-1-oxo-1-(2,3,4,5,6-pentahydroxyhexoxy)decan-5-yl]oxy-1-oxodecan-5-yl] 3,5-dihydroxydecanoate	Lipid	*Aureobasidium pullulans*	antibacterial		[19]
PI9-6	723.4533	8.1025		$C_{36}H_{68}O_{15}$	[M-H_2O+H]$^+$	109.1018, 151.1125, 297.1706, 133.1017	3-hydroxy-1-[3-hydroxy-1-oxo-1-(2,3,4,5,6-pentahydroxyhexoxy)decan-5-yl]oxy-1-oxodecan-5-yl] 3,5-dihydroxydecanoate	Lipid	*Aureobasidium pullulans*	antibacterial		[19]

PI9-7	559.384	9.1386		$C_{30}H_{56}O_{10}$	[M-H$_2$O+H]	109	5-[5-(3,5-dihydroxy-decanoyloxy)-3-hydroxydecanoyl]oxy-3-hydroxydecanoic acid	Lipid				PubChem [Internet]. Bethesda (MD): National Library of Medicine (US), National Center for Biotechnology Information; 2004-. PubChem Compound Summary for CID 45359175; [cited 2022 July 19]. Available from: https://pubchem.ncbi.nlm.nih.gov/compound/45359175
PI9-7	388.2096	10.8918	1	$C_{20}H_{31}NO_5$	[M+Na]+	109.1015, 243.0533, 127.1120, 217.0169	Brefeldin A; 7-*O*-(*N*,*N*-Dimethylaminoacetyl)		*Penicillium* sp		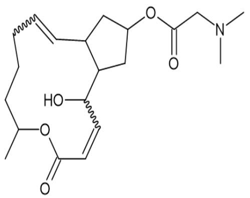	[20]
PI9-8	399.1947	8.1025	-0.5	$C_{20}H_{40}O_6$	[M+Na]+	227.1258, 238.1479	3,6,8,11-Tetrahydroxy-16,17-dimethyloctadecanoic acid	Lipid	Marine *Streptomyces* sp.	antibacterial	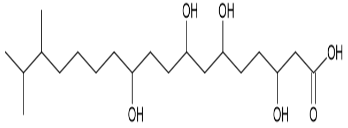	[21]
PI9-9	615.3717	7.2118	-0.5	$C_{30}H_{56}O_{11}$	[M+Na]+	429.2458, 243.1213, 395.24, 209.116, 411.2351	9-Hydroxy-exophilin A		marine *Fusarium* sp.	algicide	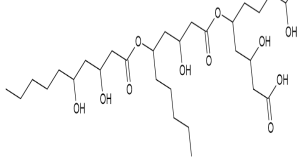	[22]
PI9-10	611.3404	7.7767	-4.1	$C_{32}H_{50}O_{11}$	[M+H]+	221.0797, 413.2508, 209.1174	WF 17819		*Ochroconis* sp.		Struct. unknown	[23]

Supplementary References

1. Shimojima, Y.; Hayashi, H.; Ooka, T.; Shibukawa, M.; Iitaka, Y. Studies on AI-77s, microbial products with gastroprotective activity. Structures and the chemical nature of AI-77s. *Tetrahedron* **1984,** *40*, 2519-2527.doi: https://doi.org/10.1016/S0040-4020(01)83504-3.
2. Berrue, F.; Ibrahim, A.; Boland, P.; Kerr, R.G. Newly isolated marine *Bacillus pumilus* (SP21): A source of novel lipoamides and other antimicrobial agents. *Pure Appl. Chem.* **2009,** *81*, 1027-1031.doi: doi:10.1351/PAC-CON-08-09-25.
3. Sugawara, T.; Tanaka, A.; Imai, H.; Nagai, K.; Suzuki, K. YM-47515, a novel isonitrile antibiotic from *Micromonospora echinospora* subsp. *echinospora*. *J. Antibiot. (Tokyo)* **1997,** *50*, 944-8.doi: 10.7164/antibiotics.50.944.
4. Zafrir Ilan, E.; Torres, M.R.; Prudhomme, J.; Le Roch, K.; Jensen, P.R.; Fenical, W. Farnesides A and B, sesquiterpenoid nucleoside ethers from a marine-derived *Streptomyces* sp., strain CNT-372 from Fiji. *J. Nat. Prod.* **2013,** *76*, 1815-8.doi: 10.1021/np400351t.
5. Costa, M.; Zuniga, P.; Penalver, A.M.; Thorsteinsdottir, M.; Perez, M.; Canedo, L.M.; Cuevas, C. New fluvirucinins C1 and C2 produced by a marine derived actinomycete. *Nat. Prod. Commun.* **2017,** *12*, 679-682.doi:
6. Itoh, J.; Omoto, S.; Nishizawa, N.; Kodama, Y.; Inouye, S. Chemical structures of amicoumacins produced by *Bacillus pumilus*. *Agric. Biol. Chem.* **1982,** *46*, 2659-2665.doi: 10.1080/00021369.1982.10865491.
7. Bruns, H.; Ziesche, L.; Taniwal, N.K.; Wolter, L.; Brinkhoff, T.; Herrmann, J.; Muller, R.; Schulz, S. N-Acylated amino acid methyl esters from marine Roseobacter group bacteria. *Beilstein J. Org. Chem.* **2018,** *14*, 2964-2973.doi: 10.3762/bjoc.14.276.
8. Vértesy, L.; Fehlhaber, H.-W.; Kogler, H.; Schindler, P.W. Enkastines: Amadori Products with a Specific Inhibiting Action against Endopeptidase – 24.11 – from *Streptomyces albus* and by Synthesis. *Liebigs Annalen* **1996,** *1996*, 121-126.doi: https://doi.org/10.1002/jlac.199619960120.
9. Ram, H.; Kumar Sahu, A.; Said, M.S.; Banpurkar, A.G.; Gajbhiye, J.M.; Dastager, S.G. A novel fatty alkene from marine bacteria: A thermo stable biosurfactant and its applications. *J. Hazard. Mater.* **2019,** *380*, 120868.doi: 10.1016/j.jhazmat.2019.120868.
10. Isono, K.; Nagatsu, J.; Kobinata, K.; Sasaki, K.; Suzuki, S. Studies on polyoxins, Antifungal Antibiotics. *Agric. Biol. Chem.* **1967,** *31*, 190-199.doi: 10.1080/00021369.1967.10858788.
11. Ding, N.; Jiang, Y.; Han, L.; Chen, X.; Ma, J.; Qu, X.; Mu, Y.; Liu, J.; Li, L.; Jiang, C.; Huang, X. Bafilomycins and odoriferous sesquiterpenoids from *Streptomyces albolongus* isolated from *Elephas maximus* Feces. *J. Nat. Prod.* **2016,** *79*, 799-805.doi: 10.1021/acs.jnatprod.5b00827.
12. Zhang, J.; Jiang, Y.; Cao, Y.; Liu, J.; Zheng, D.; Chen, X.; Han, L.; Jiang, C.; Huang, X. Violapyrones A-G, alpha-pyrone derivatives from *Streptomyces violascens* isolated from *Hylobates hoolock* feces. *J. Nat. Prod.* **2013,** *76*, 2126-30.doi: 10.1021/np4003417.
13. Cvejic, J.H.; Bodrossy, L.; Kovacs, K.L.; Rohmer, M. Bacterial triterpenoids of the hopane series from the methanotrophic bacteria *Methylocaldum* spp.: phylogenetic implications and first evidence for an unsaturated aminobacteriohopanepolyol. *FEMS Microbiol. Lett.* **2000,** *182*, 361-5.doi: 10.1111/j.1574-6968.2000.tb08922.x.
14. Kawahara, K.; Kuraishi, H.; Zähringer, U. Chemical structure and function of glycosphingolipids of *Sphingomonas* spp and their distribution among members of the α-4 subclass of Proteobacteria. *J. Ind. Microbiol. Biotechnol.* **1999,** *23*, 408-413.doi: 10.1038/sj.jim.2900708.
15. Harrigan, G.G.; Luesch, H.; Yoshida, W.Y.; Moore, R.E.; Nagle, D.G.; Biggs, J.; Park, P.U.; Paul, V.J. Tumonoic acids, novel metabolites from a cyanobacterial assemblage of *Lyngbya majuscula* and *Schizothrix calcicola*. *J. Nat. Prod.* **1999,** *62*, 464-7.doi: 10.1021/np980460u.
16. Roggo, B.E.; Petersen, F.; Delmendo, R.; Jenny, H.B.; Peter, H.H.; Roesel, J. 3-Alkanoyl-5-hydroxymethyl tetronic acid homologues and resistomycin: new inhibitors of HIV-1 protease. I. Fermentation, isolation and biological activity. *J. Antibiot. (Tokyo)* **1994,** *47*, 136-42.doi: 10.7164/antibiotics.47.136.
17. Huang, H.; Yao, Y.; He, Z.; Yang, T.; Ma, J.; Tian, X.; Li, Y.; Huang, C.; Chen, X.; Li, W.; Zhang, S.; Zhang, C.; Ju, J. Antimalarial beta-carboline and indolactam alkaloids from *Marinactinospora thermotolerans*, a deep sea isolate. *J. Nat. Prod.* **2011,** *74*, 2122-7.doi: 10.1021/np200399t.

18. Christner, C.; Kullertz, G.; Fischer, G.; Zerlin, M.; Grabley, S.; Thiericke, R.; Taddei, A.; Zeeck, A. Albocycline- and carbomycin-type macrolides, inhibitors of human prolyl endopeptidases. *J. Antibiot. (Tokyo)* **1998,** *51*, 368-71.doi: 10.7164/antibiotics.51.368.
19. Bischoff, K.M.; Leathers, T.D.; Price, N.P.; Manitchotpisit, P. Liamocin oil from Aureobasidium pullulans has antibacterial activity with specificity for species of Streptococcus. *J. Antibiot. (Tokyo)* **2015,** *68*, 642-645.doi: 10.1038/ja.2015.39.
20. Klausner, R.D.; Donaldson, J.G.; Lippincott-Schwartz, J. Brefeldin A: insights into the control of membrane traffic and organelle structure. *J. Cell Biol.* **1992,** *116*, 1071-1080.
21. Viegelmann, C.; Margassery, L.M.; Kennedy, J.; Zhang, T.; O'Brien, C.; O'Gara, F.; Morrissey, J.P.; Dobson, A.D.; Edrada-Ebel, R. Metabolomic profiling and genomic study of a marine sponge-associated *Streptomyces* sp. *Mar. Drugs* **2014,** *12*, 3323-51.doi: 10.3390/md12063323.
22. Chen, C.; Imamura, N.; Nishijima, M.; Adachi, K.; Sakai, M.; Sano, H. Halymecins, new antimicroalgal substances produced by fungi isolated from marine algae. *J. Antibiot. (Tokyo)* **1996,** *49*, 998-1005.
23. UK Patent 2 293 379 (isol, ir, pmr, cmr, props). 1996.
